# Novel Effective Fluorinated Benzothiophene-Indole Hybrid Antibacterials against *S. aureus* and MRSA Strains

**DOI:** 10.3390/ph15091138

**Published:** 2022-09-13

**Authors:** Marius Seethaler, Tobias Hertlein, Elisa Hopke, Paul Köhling, Knut Ohlsen, Michael Lalk, Andreas Hilgeroth

**Affiliations:** 1 Institute of Pharmacy, Martin Luther University Halle-Wittenberg, 06120 Halle, Germany; 2 Institute of Molecular Infection Biology, Julius Maximilians University Würzburg, 97080 Würzburg, Germany; 3 Institute of Biochemistry, Ernst Moritz Arndt University Greifswald, 17489 Greifswald, Germany

**Keywords:** antibacterial drug resistance, structure activity, synthesis, inhibition, substituent

## Abstract

Increasing antibacterial drug resistance threatens global health, unfortunately, however, efforts to find novel antibacterial agents have been scaled back by the pharmaceutical industry due to concerns about a poor return on investment. Nevertheless, there is an urgent need to find novel antibacterial compounds to combat antibacterial drug resistance. The synthesis of novel drugs from natural sources is mostly cost-intensive due to those drugs’ complicated structures. Therefore, it is necessary to find novel antibacterials by simple synthesis to become more attractive for industrial production. We succeeded in the discovery of four antibacterial compound (sub)classes accessible in a simple one-pot reaction based on fluorinated benzothiophene-indole hybrids. They have been evaluated against various *S. aureus* and MRSA strains. Structure- and substituent-dependent activities have been found within the (sub)classes and promising lead compounds have been identified. In addition, bacterial pyruvate kinase was found to be the molecular target of the active compounds. In conclusion, simple one-pot synthesis of benzothiophene-indoles represents a promising strategy for the search of novel antimicrobial compounds.

## 1. Introduction

Bacterial resistance to antibiotics is a long-established phenomenon, but it has not impacted therapy because new antibacterial drugs were constantly being discovered and made widely available [1]. Until the end of the last century, antibiotics of last resort that showed a broad antibacterial activity could be used safely for problematic strains [1]. However, there are hardly any therapy options available for a number of multi-resistant pathogens. [1,2]. The situation is further aggravated because resistance to one antibiotic usually causes cross-resistance to all antibiotics of the same class [3,4].

Various factors contribute to the increasing development of resistance [1,3]. Antibiotic misuse is the most important one. This means the use of antibiotics of last resort in cases where standard antibiotics would have been sufficiently effective, or the use of antibiotics even in instances of viral infections [1,3,5]. Moreover, the misuse in agriculture to enhance weight and prevent animal infections is still widespread worldwide [1,3,6,7]. That misuse reinforces the development of bacterial resistance as well as environmental contamination with antibiotics, which is enhanced by production companies disposing of their antibiotic production waste in rivers and oceans [1,3,7,8].

While spontaneous mutations in single bacteria may result in antibacterial drug resistance, the horizontal gene transfer of antibiotic resistance genes between various bacterial species mainly contributes to the spreading of resistance [9]. Once antibiotic resistance to an antibiotic has developed, related antibiotics of the same class or even other classes are often also ineffective due to the development of cross-resistance. Despite this long-understood phenomenon, most recently approved antibiotics have been structurally changed variants of well-established antibiotic classes. Although these compounds were developed to overcome resistant strains, there is a high risk that novel resistance develops quickly, thereby limiting the time that it can be used [10,11]. However, the development of resistance is not limited to common antibiotics; it also affects new developments with a novel mode of action, such as linezolid and daptomycin. Resistant strains were already detectable shortly after their market launch. Consequently, there is a strong need for permanent development of new substances in order to have antibiotics of last resort available at any time. In contrast to the urgent need for new antibacterial drugs, a number of pharmaceutical companies have stopped their efforts to develop new antibiotics in the last two decades because they consider this market segment to be unprofitable [3,12]. The costs for a successful drug development are independent from the indication, but drugs to treat chronic diseases or lifestyle drugs are more profitable than a curative antibiotic that would only used only for a short period. Moreover, antibiotics should be available at low costs, especially in developing countries, where low hygiene standards afford a broader antibiotic use due to infections with pathogens [1,13,14,15].

However, it may be attractive to find novel antibiotics using simple, low-cost procedures for industrial production, in contrast to the mostly cost-intensive synthesis of antibiotics from natural sources with complicated molecular structures [16,17].

**Figure 1:**
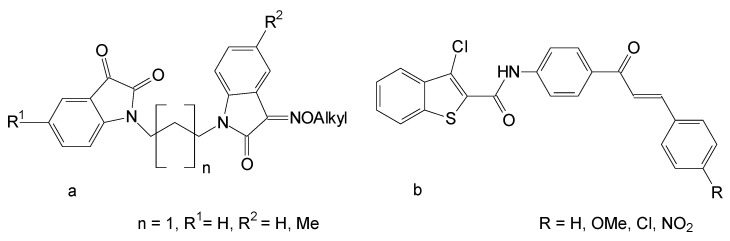
Structures of bisindoles with the indole scaffold (**a**) and the benzothiophenes with shown scaffold (**b**).

Earlier classes of antibacterial compounds have been bisindoles with an indole scaffold and benzothiophenes with a benzothiophene scaffold for that low to moderate antimicrobial activities have been reported (Figure 1) [18,19]. 

We found novel antibacterial compound (sub)classes in a simple one-pot reaction with structurally varied substitution patterns within the molecular scaffold, and combined them with substituted indole residues at respective positions of the scaffold. Structurally, the scaffold is composed of an indole that is attached to a benzothiophene, with the thiophene sulfur being oriented at the lower and the upper molecular scaffold site. Together with the differently positioned indole residue, either at the upper or the lower molecular scaffold site, we found novel hybrid antibiotics with promising effects for enhanced antibacterial activities.

We evaluated our four compound (sub)classes against *S. aureus* and MRSA strains to prospectively counteract MRSA drug resistance. 

## 2. Results and Discussion

### 2.1. Formation of the Substituted Indolobenzothiophenes

**Scheme 1:**
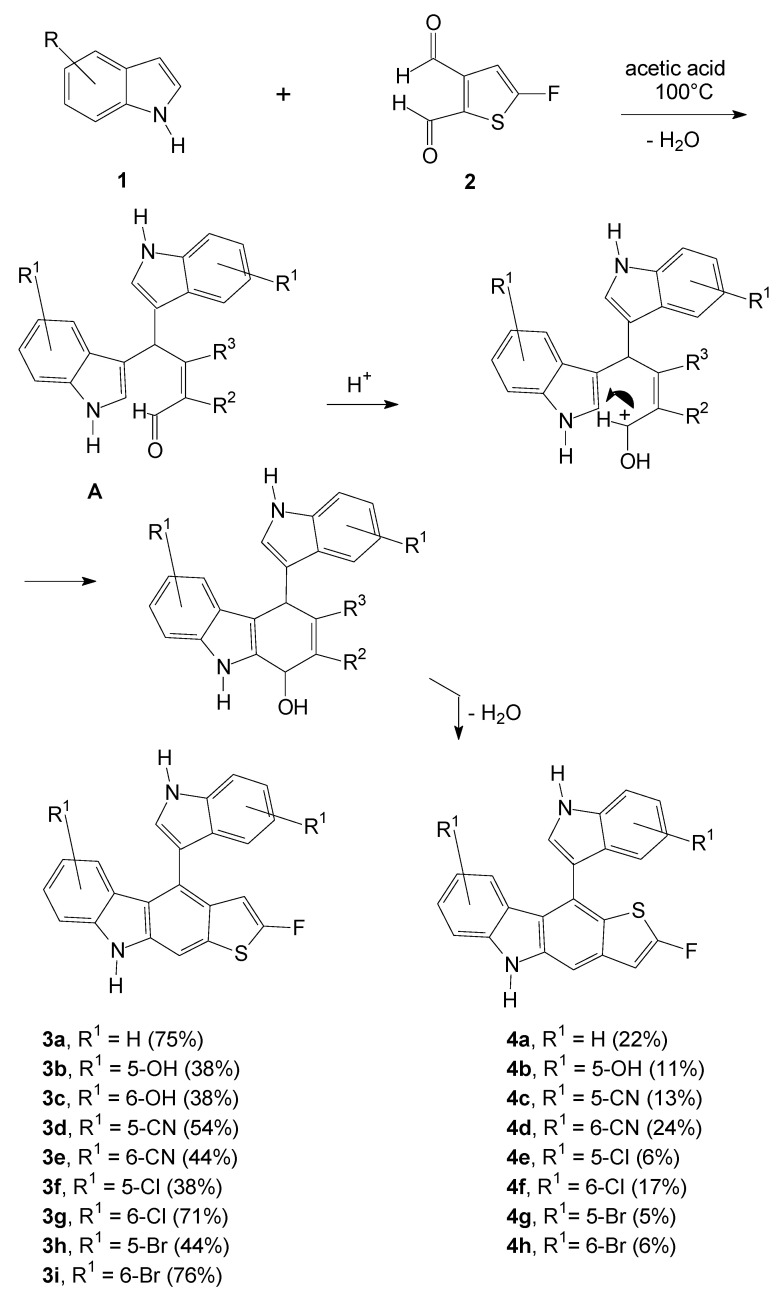
Formation of the 5-Fluoro– indolobenzothiophenes **3** and **4** with yields in brackets.

Generally, in hybrid antibiotics, both subunits with known antibacterial activities are connected by a flexible linker chain [20,21]. In our hybrid molecules, the indole and the benzothiophene subunits are directly connected by the reaction of one aldehyde function of the 5-fluorothiophene-2,3-dicarbaldehyde **2** with the nucleophilic 3-position of one indole **1** (Scheme 1). 

**Scheme 2:**
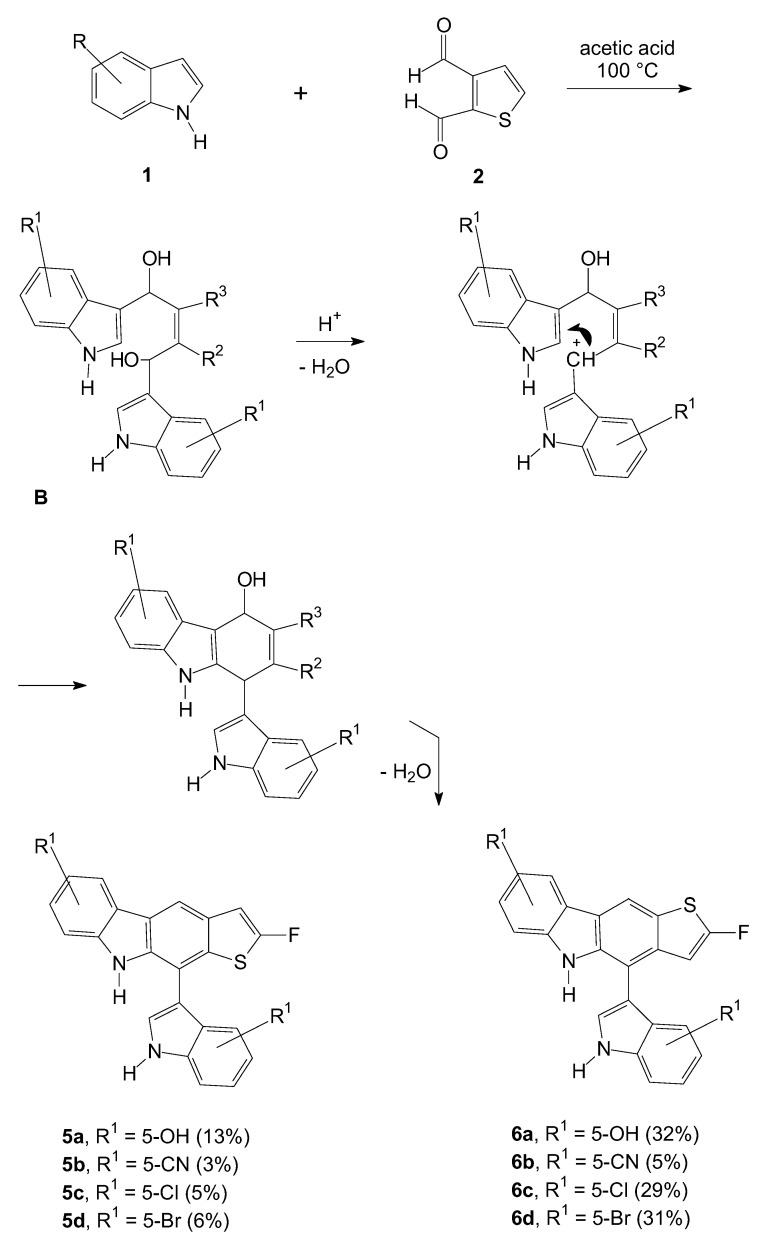
Formation of the 5-Fluoroi– indolobenzothiophenes **5** and **6** with yields in brackets.

Intermediate **A** results from the reaction of one aldehyde function of starting compound **2** with each the nucleophilic 3-position of two indoles **1** under water elimination to form the bisindolyl core of intermediate **A**. After attack of the aldehyde function of **A** to the 2-position of the first reacted indole and a final elimination of a water molecule, the compound subclasses **3** and **4** are yielded. The compound subclasses **5** and **6** result from a primary attack of the nucleophilic indole 3-position of two indoles to each one aldehyde function of starting compound **2** to give intermediate **B** (Scheme 2). Then, a water elimination and a carbenium attack to the 2-position of the first attacked indole and a final water elimination lead to both aromatized compounds **5** and **6**. Thus, we finally reach directly connected indolobenzothiophenes with one additional indole attached to the upper molecule site or the lower molecule site in a one-pot reaction.

The reaction proceeding of indole **1** and 5-fluorothiophene-2,3-dicarbaldehyde **2** at 100 °C in acetic acid was followed by thin layer chromatography, and after a work-up procedure and compound extraction, the target compounds **3**–**6** were isolated by column chromatography over silica gel in the resulting compound fractions that were evaporated, and from that, the compounds were finally crystallized. 

### 2.2. Antibacterial S. aureus and MRSA Activities of the Indolobenzothiophenes

The antibacterial activity was determined as MIC (minimal inhibitory concentration) value, meaning that at the given concentration, no bacterial growth could be observed. The two-fold serial dilution technique was applied for the determination starting with stock solutions in DMSO. Oxacillin and ciprofloxacin have been used as standard antibiotics. First, the compound activity was determined in the MRSA strain USA300 Lac * lux. In case of an activity < 16 µg/mL, the activity against the MRSA strain JE2 and two methicillin-sensitive (MSSA) standard strains, ATCC6538 and HG003, was determined. Table 1 shows the compound activity against the MRSA and MSSA strains tested. 

In compound class **3**, we determined a MIC value of 1 µg/mL for the indole unsubstituted derivative **3a** in the USA Lac * lux strain, and of 2 µg/mL in the MRSA JE2 strain.

Thus, the compound was twenty-four-fold more active than the used standard antibiotic oxacillin, and thirty-two-fold more active than ciprofloxacin. In the MSSA strains, we found an almost similar activity that was in the range of that determined for both ciprofloxacin and oxacillin.

A 5-hydroxy indole function in compound **3b** decreased the activity that was found to be similar in the MRSA and the MSSA strains with a MIC value of 8 µg/mL. If that hydroxyl function moved to the 6-position of the indole in compound **3c**, the activity was found to be improved, with a mean value of 2 µg/mL in both the MRSA and the MSSA strains. Therefore, the 6-indole substitution tends to be more favourable than the 5-indole positioning.

A 5-cyano substitution in derivative **3d** was much more favourable than the 5-hydroxy function, with values of 0.75 µg/mL in the MRSA strains USA Lac * lux and JE2. Thus, compound **3d** was more active than the indole-unsubstituted compound **3a.** Additionally, in both MSSA strains HG003 and ATCC6538, we found increased activities compared to the indole-unsubstituted compound **3a**.

For the 6-cyano compound **3e** we determined almost the same activities in the MRSA strains compared to the 5-cyano derivative **3d**. In the MSSA strains both activities were almost similar compared to compound **3d**. 

The 5-chloro substitution in derivative **3f** resulted in a decreased activity compared to the 5-cyano substitution in compound **3d** in USA Lac * lux and the MRSA strain JE2. Additionally, the MSSA activities were found to be decreased compared to the 5-cyano-substituted compound **3d**.

If the chloro substitution moved to the 6-position in compound **3g**, the MRSA activities were found to be slightly increased, whereas the MSSA activities remained almost unchanged.

The 5-bromo substitution in compound **3h** was less favourable in one MSSA strain compared to the 5-chloro substitution in derivative **3f**, whereas the MRSA activities were found unchanged. The 6-bromo substitution in compound **3i** was less favourable than the 5-bromo substitution in both MRSA strains, and slightly improved activities were found for one MSSA strain.

For compound class **3**, it can be generally stated that a substitution of the 6-position is more favourable than a 5-substituion.

In compound class **4**, the sulfur of the thiophene ring is located at a different position, at the molecular site near to the attached indole residue. The indole-substituted compound **4a** showed an antibacterial activity towards both MRSA and MSSA strains almost identical to compound **3a**. The 5-hydroxy indole substitution of derivative **4b** was just slightly more favourable in the MRSA strains and less favourable in the MSSA strains, as compared to compound **3b**. 

The 5-cyano function activity in compound **4c** differed in the respective MRSA and MSSA strains. In the USA300 Lac * lux strain, the activity was almost similar to compound **3d**. In strain JE2, it was found to be mainly decreased, with a MIC of 24 µg/mL. In the MSSA strain HG003, the activity was also lower compared to that in strain ATCC6538, with a MIC value similar to compound **3d**. If the cyano function moved to the 6-position of the indole residue in compound **4d**, the antibacterial activity was found to be generally increased. While the activity in the USA300 Lac * lux was found to be slightly increased compared to compound **4c**, the activity towards JE2 was twenty-four-fold higher, with a MIC value of 1 µg/mL, which was identical to derivative **3e**. In the MSSA strain HG003, it was found to be four-fold increased compared to compound **4c** and also better in the strain ATCC6538. Compared to derivative **3e** the activity in ATCC6538 was also improved.

The activity of the 5-chloro indole compound **4e** was found unchanged in the MRSA strains compared to that of compound **3f**, but in the MSSA strains, it was found decreased compared to **3f**.

A movement of the chloro indole function to the 6-position resulted in an unchanged activity of derivative **4f** in the MRSA strains, whereas the activity in the MSSA strains was found increased also in comparison to compound **3g**.

The 5-bromo substitution in derivative **4g** resulted in a lowered activity in the MRSA strains as compared to compound **3h**. However, the activity in both MSSA strains was found to be increased compared to that of **3h**. The 6-bromo function in compound **4h** resulted in an almost unchanged activity in the USA300 Lac * lux strain, whereas that towards JE2 was found to be increased. In the MSSA strains we found an unchanged activity towards HG003 and a decreased activity towards ATCC6538.

Similar to compound class **3**, the substitution of the 6-position in class **4** is more favourable than the 5-position and the hydroxy indole substitution was as unfavourable, as in compound class **3**. 

In compound class **5**, the indole substituent is placed at the lower site of the molecular scaffold with the same position of the sulfur in the thiophene ring, as in compound class **3**.

Surprisingly, the 5-hydroxy indole derivative **5a** had an improved activity towards both the MRSA and the MSSA strains compared to derivative **3b**, with the indole placed at the upper molecular site. If that hydroxy function was replaced with a cyano function in compound **5b**, the activity was found to be mainly reduced. We just found a residual activity with a MIC value of 64 µg/mL in the USA Lac * lux strain. This is surprising again, because the corresponding compound **3d** with the indole residue at the upper molecular site had an impressive activity, with the best results within that compound class. The 5-chloro function in derivative **5c** resulted in increased activities that were not as good in the MSSA strains compared to those of the 5-hydroxy indole compound **5a**. The 5-bromo indole substitution in compound **5d** was less favourable than the 5-chloro indole substitution. Both activities were found to be reduced, with that of the MSSA HG003 being lowest. Interestingly, that activity was also the lowest of the MRSA and MSSA activities of corresponding compound **3h**.

Last, we characterized the antibacterial activities of compound class **6**. The 5-hydroxy indole substitution of derivative **6a** was as favourable as that of the related compound **5a**, with the different sulfur positioning in the MRSA strains. However, in the MSSA strains, the activity was decreased. However, if compared to compound **4b** with the indole residue placed at the upper molecule site, the activity is improved in both the MRSA and the MSSA strains. The replacement of the hydroxyl with the cyano function in derivative **6b** lowered the activity, but the extent was less than that for compound **5b** in the MRSA strain USA LAC * lux. The 5-chloro indole substitution of compound **6c** again showed an increased activity similar to the 5-hydroxy indole derivative **6a**. The 5-bromo indole substitution in compound **6d** was, again, less favourable, but similar to that of the related compound **5d,** with differences in the MSSA strain activities.

The 5-hydroxy indole-substituted compounds of both classes **5** and **6** showed the best antibacterial activities, whereas this substitution was less favourable in compound classes **3** and **4**. Whereas the 5-cyano indole substitution resulted in the best activities, especially in compound **3d**, the 5-cyano indole substitution showed the lowest results in compounds **5b** and **6b**.

**Figure 2:**
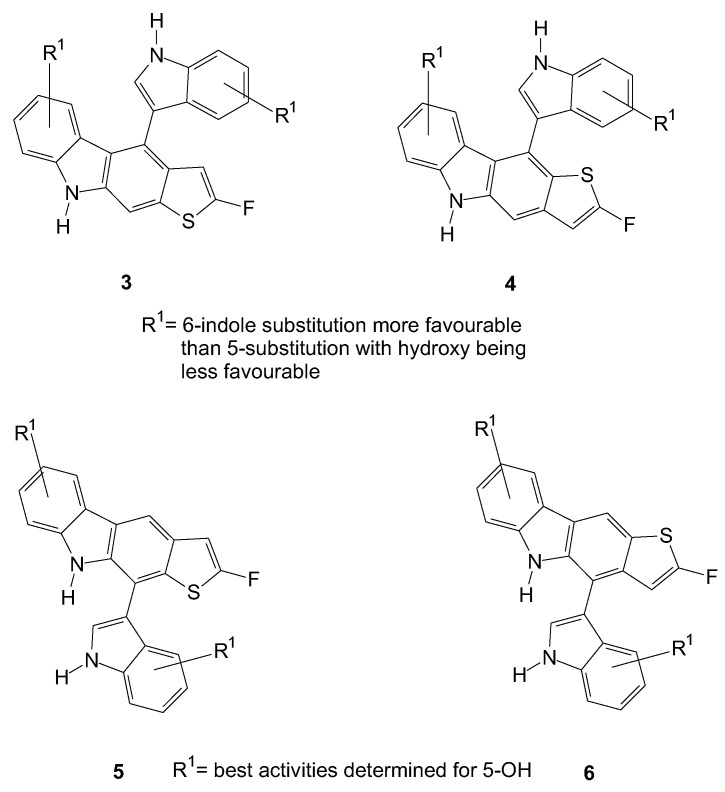
SAR shown for compound classes **3**–**6**.

The structure–activity relationships (SAR) are summarized in Figure 2. 

In our antibacterial compound classes, the two indoles are neighboured, with one being part of the molecular scaffold and the other being attached either at the upper molecular site or the lower molecular site. Such indoles related to one another have also been found in the marine alkaloids spongotine, topsentin, and hamacanthin, with antibacterial activity towards MRSA in micromolar ranges that were reported to inhibit the MRSA pyruvate kinase (PK) in submicromolar ranges [22]. 

To test if the anti-*S. aureus* activity of our compounds may also be linked to inhibition of PK, we selected representative compounds of our compound classes and determined the inhibition of MRSA pyruvate kinase that was overexpressed in *Escherichia coli*. Following purification, PK activity was determined using a luminescence-based assay that detects ATP generation from conversion of phosphoenolpyruvate to pyruvate by PK. Due to an observed low stability of PK, only selected compounds could be characterized. In this assay system, we determined that the two selected compounds **3c** and **3f** with the lowest MRSA and MSSA activities with mean values of 2.25 µg/mL and 2.75 µg/mL resulted in IC_50_ values of PK inhibition of 1.6 µM and 2.1 µM, respectively. For compound **4f**, with an mean antibacterial activity of 3 µg/mL, we determined an IC_50_ value of 2.3 µM. Compound **6d** with a mean antibacterial activity of 10 µg/mL resulted in a IC_50_ value of 3.5 µM. Compounds **5b** and **6b**, with residual activities towards MRSA (≥32 µg/mL), were not active as PK inhibitors.

## 3. Material and Methods

### 3.1. Chemical Reagents and Instruments

Commercial reagents were used without further purification. The ^1^H-NMR spectra (500 MHz) were measured using tetramethylsilane as internal standard using a DD2 instrument of Agilent Technologies. The following abbreviations have been used for characterization: singlet (s), doublet (d), doublet of doublet (dd), doublet from doublet from doublet (ddd), triplet (t), septet (sept) and broad (br). The spectra are shown in the Supplementary Materials. Thin layer chromatography (TLC) was performed on E. Merck 5554 silica gel plates. The high-resolution mass spectra were recorded on a Finnigan LCQ Classic mass spectrometer. 

### 3.2. General Procedure for the Synthesis of Compounds ***3***–***6***

Two mmol of the respective indole **1** in milligram, depending on the molecular weight, and one mmol (165 mg) of the 5-fluorothiophene-2,3-dicarbaldehyde **2** were dissolved in 15 mL of acetic acid. The mixture was heated at 100 °C under reflux for at least 2 h. The preceding reaction was followed by TLC, using mixtures of ethyl acetate and cyclohexane. After completion of the reaction, the mixture was cooled down to room temperature and neutralized with sodium hydroxide solution (2.5 M). Then, it was thrice extracted with ethyl acetate (20 mL). The unified organic layer was dried over sodium sulfate for 1 h, filtered, and removed in a vacuum. The oily residue was purified by column chromatography over silica gel, and from the resulting compound fractions, the eluent was removed again in a vacuum to produce products **3**–**6** as powders. The eluents were the same as those used for the TLC, with ethyl acetate and cyclohexane mixtures of 22:78 for compounds **3a** and **4a**, 25:75 for compounds **3g**, **3i**, **4f** and **4h**, 33:67 for compounds **3f**, **3h**, **4e**, **4g**, **5c**, **5d**, **6c** and **6d**, 50/50 for compounds **3b**, **3c**, **3e**, **4b**, **4d**, **5a** and **6a,** and 67/33 for compounds **3d**, **4c**, **5b** and **6b**. 

*2-Fluoro-4-(1H-indol-3-yl)-9H-thieno [2,3-b]carbazole (**3a**)*. Yield 73%, decomposition 239 °C, white powder; ^1^H NMR (dmso-*d*_6_) *δ* = 11.52 (s_br_, *^3^J_NH′/2′_*, 1H, N′-H), 11.37 (s, 1H, N-H), 7.97 (s, 1H, 10-H), 7.64 (d, *^3^J_2′/NH′_* = 2.4 Hz, 1H, 2′-H), 7.57 (d“t”, *^3^J_7′/6′_* = 8.2, *^4^J_7′/5′_* = 1.0 Hz, 1H, 7′-H), 7.43 (d“t”, *^3^J_8/7_* = 8.2, *^4^J_8/6_* = 1.1 Hz, 1H, 8-H), 7.25 (“sept”, *^3^J_7/8_* = 8.2, *^3^J_7/6_* = 7.1, *^4^J_7/5_* = 1.2 Hz, 1H, 7-H), 7.17 (“sept”, *^3^J_6′/7′_* = 8.2, *^3^J_6′/5′_* = 6.8, *^4^J_6′/4′_* = 1.2 Hz, 1H, 6′-H), 6.98 (d_br_, *^3^J_5/6_* = 8.1 Hz, *^4^J_5/7_*, 1H, 5-H), 6.97 (d_br_, *^3^J_4′/5′_* = 7.9 Hz, *^4^J_4′/6′_*, 1H, 4′-H), 6.91 (“sept”, *^3^J_5′/4′_* = 7.9, *^3^J_5′/6′_* = 6.8, *^4^J_5′/7′_* = 1.0 Hz, 1H, 5′-H), 6.73 (“sept”, *^3^J_6/5_* = 8.1, *^3^J_6/7_* = 7.1, *^4^J_6/8_* = 1.1 Hz, 1H, 6-H), 6.60 (d, *^3^J* (^1^H,^19^F) = 3.2 Hz, 1H, 3-H); ^13^C-NMR (dmso-*d*_6_) *δ* = 167.3 (d), 141.3, 137.7 (d), 137.4, 131.8, 130.6, 129.7 (d), 126.4, 124.9, 124.7, 122.0, 122.6, 121.8, 121.4, 121.3, 119.8, 119.7, 111.1, 111.0, 104.6 (d); *m/z* (ESI, %) 355.0709 (27, [C_22_H_12_FN_2_S]^−^ − 1.5907 ppm); 391.0475 (100, [C_22_H_13_ClFN_2_S]^−^ − 0.7560 ppm).

*2-Fluoro-4-(5-hydroxy-1H-indol-3-yl)-9H-thieno[2,3-b]carbazol-6-ol (**3b**)*. Yield 38%, mp. 192–195 °C, pale yellow powder; ^1^H NMR (dmso-*d*_6_) *δ* = 11.18 (d, *^3^J_NH′/2′_* = 2.5 Hz, 1H, N′-H), 10.97 (s, 1H, N-H), 8.56 (s, 1H, O-H), 8.50 (s, 1H, O′-H), 7.85 (s, 1H, 10-H), 7.46 (d, *^3^J_2′/NH′_* = 2.5 Hz, 1H, 2′-H), 7.34 (d, *^3^J_7′/6′_* = 8.7 Hz, 1H, 7′-H), 7.21 (d, *^3^J_8/7_* = 8.6 Hz, 1H, 8-H), 6.78 (dd, *^3^J_7/9_* = 8.6, *^4^J_7/5_* = 2.4 Hz, 1H, 7-H), 6.66 (dd, *^3^J_6′/7′_* = 8.7, *^4^J_6′/4′_* = 2.2 Hz, 1H, 6′-H), 6.57 (d, *^4^J_5/7_* = 2.4 Hz, 1H, 5-H), 6.49 (d, *^3^J* (^1^H,^19^F) = 3.0 Hz, 1H, 3-H), 6.33 (d, *^4^J_4′/6′_* = 2.2 Hz, 1H, 4′-H); ^13^C-NMR (dmso-*d*_6_) *δ* = 167.3 (d), 152.4, 152.3, 137.6 (d), 133.9, 131.8, 130.0, 129.9, 129.8 (d), 129.7, 124.9, 124.7, 122.8, 122.6, 112.8, 112.7, 112.5, 112.4, 110.1, 106.7, 106.6, 104.6 (d); *m/z* (ESI, %) 423.0374 (100, [C_22_H_13_ClFN_2_O_2_S]^−^ − 0.4874 ppm).

*2-Fluoro-4-(6-hydroxy-1H-indol-3-yl)-9H-thieno[2,3-b]carbazol-7-ol (**3c**).* Yield 38%, mp. 179–182 °C, white greyish yellow powder; ^1^H NMR (dmso-d_6_) *δ* = 11.04 (d, *^3^J_NH′/2′_* = 2.3 Hz, 1H, N′-H), 11.03 (s, 1H, N-H), 9.31 (s, 1H, O-H), 8.97 (s, 1H, O′-H), 7.80 (s, 1H, 10-H), 7.34 (d, *^3^J_2′/NH′_* = 2.3 Hz, 1H, 2′-H), 6.89 (dd, *^4^J_7′/5′_* = 2.1 Hz, 1H, 7′-H), 6.83 (d, *^3^J_5/6_* = 8.6 Hz, 1H, 5-H), 6.75 (d, *^4^J_8/6_* = 2.2 Hz, 1H, 8-H), 6.73 (d, *^3^J_4′/5′_* = 8.5 Hz, 1H, 4′-H), 6.58 (d, *^3^J* (^1^H,^19^F) = 3.3 Hz, 1H, 3-H) 6.44 (dd, *^3^J_5′/4′_* = 8.5, *^4^J_5′/7′_* = 2.1 Hz, 1H, 5′-H), 6.22 (dd, *^3^J_6/5_* = 8.6, *^4^J_6/8_* = 2.2 Hz, 1H, 6-H); ^13^C-NMR (dmso-*d*_6_) *δ* = 167.2 (d), 156.9, 156.8, 143.4, 137.7 (d), 137.6, 131.8, 129.7 (d), 124.9, 124.7, 123.2, 122.8, 122.6, 122.2, 122.1, 119.0, 110.9, 110.8, 110.1, 104.6 (d), 96.8, 96.7; *m*/*z* (ESI, %) 423.0372 (100, [C_22_H_13_ClFN_2_O_2_S]^−^ − 0.8668 ppm).

*4-(5-Cyano-1H-indol-3-yl)-2-fluoro-9H-thieno[2,3-b]carbazole-6-carbonitrile (**3d**)*. Yield 54%, mp. 162–165 °C, white yellow powder; ^1^H NMR (dmso-*d*_6_) *δ* = 12.22 (s_br_, *^3^J_NH′/2′_*, 1H, N′-H), 12.09 (s, 1H, N-H), 8.17 (s, 1H, 10-H), 7.97 (d, *^3^J_2′/NH′_* = 2.5 Hz, 1H, 2′-H), 7.79 (d, *^3^J_7′/6′_* = 8.5 Hz, 1H, 7′-H), 7.67 (dd, *^3^J_7/8_* = 8.5, *^4^J_7/5_* = 1.6 Hz, 1H, 7-H), 7.61 (d, *^3^J_8/7_* = 8.5 Hz, 1H, 8-H), 7.57 (dd, *^3^J_6′/7′_* = 8.5, *^4^J_6′/4′_* = 1.5 Hz, 1H, 6′-H), 7.42 (d, *^4^J_4′/6′_* = 1.5 Hz, 1H, 4′-H), 7.11 (d, *^4^J_5/7_* = 1.6 Hz, 1H, 5-H), 6.68 (dd, *^3^J* (^1^H,^19^F) = 3.1 Hz, 1H, 3-H); ^13^C-NMR (dmso-*d*_6_) *δ* = 167.4 (d), 145.6, 141.7, 137.5 (d), 131.8, 129.7 (d), 129.2, 127.1, 125.5, 123.4, 124.9, 124.7, 123.8, 123.7, 122.8, 122.6, 118.6, 118.5, 111.8, 110.1, 104.6 (d), 101.5, 101.6; *m/z* (ESI, %) 405.0610 (100, [C_24_H_10_FN_4_S]^−^ − 1.4842 ppm).

*4-(6-Cyano-1H-indol-3-yl)-2-fluoro-9H-thieno[2,3-b]carbazole-7-carbonitrile (**3e**)*. Yield 44%, decomposition 175 °C, white powder; ^1^H NMR (dmso-*d*_6_) *δ* = 12.19 (s_br_, *^3^J_NH′/2′_*, 1H, N′-H), 11.91 (s, 1H, N-H), 8.15 (s, 1H, 10-H), 8.09 (dd, *^4^J_7′/5′_* = 1.5, *^5^J_7′/4′_* = 0.8 Hz, 1H, 7′-H), 8.02 (d, *^3^J_2′/NH′_* = 2.6 Hz, 1H, 2′-H), 7.92 (dd, *^4^J_8/6_* = 1.5, *^5^J_8/5_* = 0.7 Hz, 1H, 8-H), 7.25 (dd, *^3^J_5′/4′_* = 8.3, *^4^J_5′/7′_* = 1.5 Hz, 1H, 5′-H), 7.17 (dd, *^3^J_6/5_* = 8.3, *^4^J_6/8_* = 1.5 Hz, 1H, 6-H), 7.08 (d_br_, *^3^J_4′/5′_* = 8.3 Hz, *^5^J_4′/7′_*, 1H, 4′-H), 7.08 (d_br_, *^3^J_5/6_* = 8.3 Hz, *^5^J_5/8_*, 1H, 5-H), 6.65 (d, *^3^J* (^1^H,^19^F) = 3.3 Hz, 1H, 3-H); ^13^C-NMR (dmso-*d*_6_) *δ* = 167.5 (d), 137.0, 137.8 (d), 136.2, 134.9, 131.8, 130.7, 129.7 (d), 125.9, 125.8, 124.9, 124.7, 122.8, 122.6, 119.5, 119.4, 118.6, 118.5, 114.5, 114.4, 110.1, 104.6 (d), 99.5, 99.4; *m/z* (ESI, %) 405.0621 (100, [C_24_H_10_FN_4_S]^−^ + 1.3244 ppm).

*6-Chloro-4-(5-chloro-1H-indol-3-yl)-2-fluoro-9H-thieno[2,3-b]carbazole (**3f**)*. Yield 38%, mp. 201–204 °C, white powder; ^1^H NMR (dmso-*d*_6_) *δ* = 11.79 (s_br_, *^3^J_NH′/2′_*, 1H, N′-H), 11.59 (s, 1H, N-H), 8.05 (s, 1H, 10-H), 7.77 (d, *^3^J_2′/NH′_* = 2.5 Hz, 1H, 2′-H), 7.61 (d, *^3^J_7′/6′_* = 8.7 Hz, 1H, 7′-H), 7.46 (d, *^3^J_8/7_* = 8.6 Hz, 1H, 8-H), 7.29 (dd, *^3^J_7/8_* = 8.6, *^4^J_7/5_* = 2.2 Hz, 1H, 7-H), 7.20 (dd, *^3^J_6′/7′_* = 8.7, *^4^J_6′/4′_* = 2.1 Hz, 1H, 6′-H), 6.91 (d, *^4^J_4′/6′_* = 2.1 Hz, 1H, 4′-H), 6.85 (d, *^4^J_5/7_* = 2.2 Hz, 1H, 5-H), 6.61 (dd, *^3^J* (^1^H,^19^F) = 3.2 Hz, 1H, 3-H); ^13^C-NMR (dmso-*d*_6_) *δ* = 167.2 (d), 139.4, 137.4 (d), 135.5, 131.8, 129.9, 129.7 (d), 127.8, 125.6, 125.5, 124.9, 124.7, 122.8, 1222.6, 122.4, 122.3, 121.7, 121.6, 114.1, 114.0, 110.1, 104.3 (d); *m/z* (ESI, %) 422.9938 (100, [C_22_H_10_Cl_2_FN_2_S]^−^ + 1.5654 ppm).

*7-Chloro-4-(6-chloro-1H-indol-3-yl)-2-fluoro-9H-thieno[2,3-b]carbazole (**3g**)*. Yield 71%, mp. 128–131 °C, white powder; ^1^H NMR (dmso-*d*_6_) *δ* = 11.68 (s_br_, *^3^J_NH′/2′_* = 2.5 Hz, 1H, N′-H), 11.55 (s, 1H, N-H), 8.03 (s, 1H, 10-H), 7.71 (d, *^3^J_2′/NH′_* = 2.5 Hz, 1H, 2′-H), 7.60 (“t”, *^4^J_7′/5′_*, 1H, 7′-H), 7.46 (d, *^4^J_8/6_* = 1.9 Hz, 1H, 8-H), 6.94–6.91 (m, 2H: 4′-H, 5′-H), 6.87 (d, *^3^J_5/6_* = 8.5 Hz, 1H, 5-H), 6.80 (dd, *^3^J_6/5_* = 8.5, *^4^J_6/8_* = 1.9 Hz, 1H, 6-H), 6.63 (d, *^3^J* (^1^H,^19^F) = 3.2 Hz, 1H, 3-H); ^13^C-NMR (dmso-*d*_6_) *δ* = 167.3 (d), 137.7 (d), 137.6, 136.9, 131.8, 129.7 (d), 128.7, 128.2, 128.1, 124.9, 124.5, 124.7, 123.8, 123.7, 122.8, 122.6, 121.3, 121.2, 111.9, 111.8, 110.1, 104.6 (d); *m/z* (ESI, %) 422.9940 (100, [C_22_H_10_Cl_2_FN_2_S]^−^ + 1.9550 ppm).

*6-Bromo-4-(5-bromo-1H-indol-3-yl)-2-fluoro-9H-thieno[2,3-b]carbazole (**3h**)*. Yield 44%, mp. 239–242 °C, white powder; ^1^H NMR (dmso-*d*_6_) *δ* = 11.81 (s_br_, *^3^J_H′/2′_*, 1H, N′-H), 11.61 (s, 1H, N-H), 8.05 (s, 1H, 10-H), 7.75 (d, *^3^J_2′/NH′_* = 2.5 Hz, 1H, 2′-H), 7.57 (d, *^3^J_7′/6′_* = 8.6 Hz, 1H, (7′-H), 7.42 (d, *^3^J_8/7_* =8.6 Hz, 1H, 8-H), 7.40 (dd, *^3^J_7/8_* = 8.6, *^4^J_7/5_* = 1.7 Hz, 1H, 7-H), 7.31 (dd, *^3^J_6′/7′_* = 8.6, *^4^J_6′/4′_* = 1.9 Hz, 1H, 6′-H), 7.05 (d, *^4^J_4′/6′_* = 1.9 Hz, 1H, 4′-H), 7.01(d, *^4^J_5/7_* = 1.7 Hz, 1H, 5-H), 6.61 (dd, *^3^J* (^1^H,^19^F) = 3.2 Hz, 1H, 3-H); ^13^C-NMR (dmso-*d*_6_) *δ* = 167.5 (d), 140.3, 137.6 (d), 136.4, 131.8, 130.7, 129.7 (d), 128.6, 126.2, 126.0, 124.9, 124.7, 124.6, 124.5, 122.8, 122.6, 122.5, 122.4, 113.3, 113.2, 110.1, 104.6 (d); *m/z* (ESI, %) 512.8904 (100, [C_22_H_10_Br^81^BrFN_2_S]^−^ + 0.7000 ppm).

*7-Bromo-4-(6-bromo-1H-indol-3-yl)-2-fluoro-9H-thieno[2,3-b]carbazole (**3i**)*. Yield 76%, mp. 111–114 °C, white powder; ^1^H NMR (dmso-*d*_6_) *δ* = 11.68 (d, *^3^J_NH′/2′_* = 2.4 Hz, 1H, N′-H), 11.54 (s, 1H, N-H), 8.02 (s, 1H, 10-H), 7.75 (d, *^4^J_7′/5′_* = 1.7 Hz, 1H, 7′-H), 7.69 (d, *^3^J_2′/NH′_* = 2.4 Hz, 1H, 2′-H), 7.61 (d, *^4^J_8/6_* = 1.8 Hz, 1H, 8-H), 7.04 (dd, *^3^J_5′/4′_* = 8.5, *^4^J_5′/7′_* = 1.7 Hz, 1H, 5′-H), 6.92 (dd, *^3^J_6/5_* = 8.5, *^4^J_6/8_* = 1.8 Hz, 1H, 6-H), 6.86 (d, *^3^J_4′/5′_* = 8.5 Hz, 1H, 4′-H), 6.80 (d, *^3^J_5/6_* = 8.5 Hz, 1H, 5-H), 6.61 (d, *^3^J* (^1^H,^19^F) = 3.2 Hz, 1H, 3-H); ^13^C-NMR (dmso-*d*_6_) *δ* = 167.5 (d), 138.5, 137.1 (d), 135.2, 131.8, 129.7 (d), 129.6, 125.7, 125.6, 125.4, 124.9, 124.7, 123.2, 123.1, 122.8, 122.6, 114.9, 114.4, 114.3, 110.1, 104.6 (d); *m/z* (ESI, %) 512.8895 (100, [C_22_H_10_Br^81^BrFN_2_S]^−^ − 1.0946 ppm).

*2-Fluoro-10-(1H-indol-3-yl)-5H-thieno[3,2-b]carbazole (**4a**)*. Yield 22%, decomposition 235 °C, white powder; ^1^H NMR (dmso-*d*_6_) *δ* = 11.59 (s_br_, 1H, N′-H), 11.35 (s, 1H, N-H), 7.79 (s, 1H, 4-H), 7.75 (d, *^3^J_2′/NH′_* = 2.5 Hz, 1H, 2′-H), 7.58 (d“t”, *^3^J_7′/6′_* = 8.2, *^4^J_7′/5′_* = 1.0 Hz, 1H, 7′-H), 7.43 (d“t”, *^3^J_6/7_* = 8.1, *^5^J_6/8_* = 1.0 Hz, 1H, 6-H), 7.25 (“sept”, *^3^J_7/6_* = 8.2, *^3^J_7/8_* = 7.1, *^4^J_7/9_* = 1.2 Hz, 1H, 7-H), 7.19 (“sept”, *^3^J_6′/7′_* = 8.2, *^3^J_6′/5′_* = 6.9, *^4^J_6′/4′_* = 1.3 Hz, 1H, 6′-H), 7.18 (s, *^3^J* (^1^H,^19^F) = 3.0 Hz, 1H, 3-H), 6.99 (d_br_, *^3^J_4′/5′_* = 8.1 Hz, *^3^J_4′/6′_*, 1H, 4′-H), 6.94 (d_br_, *^3^J_9/8_* = 8.1 Hz, *^3^J_9/7_*, 1H, 9-H), 6.92 (“sept”, *^3^J_5′/4′_* = 8.1, *^3^J_5′/6′_* = 6.9, *^4^J_5′/7′_* = 1.0 Hz, 1H, 5′-H), 6.74 (“sept”, *^3^J_8/9_* = 8.1, *^3^J_8/7_* = 7.1, *^4^J_8/6_* = 1.0 Hz, 1H, 8-H); ^13^C-NMR (dmso-*d*_6_) *δ* = 167.5 (d), 141.3, 137.6 (d), 137.4, 130.6, 130.5, 128.8 (d), 126.4, 124.8, 124.7, 123.8, 122.9, 121.7, 121.6, 121.4, 121.3, 119.8, 119.7, 110.1, 111.1, 111.0, 104.6 (d); *m/z* (ESI, %) 355.0715 (27, [C_22_H_12_FN_2_S]^−^ + 1.2263 ppm); 391.0483 (100, [C_22_H_13_ClFN_2_S]^−^ − 1.3962 ppm).

*2-Fluoro-10-(5-hydroxy-1H-indol-3-yl)-5H-thieno[3,2-b]carbazol-8-ol (**4b**)*. Yield 11%, mp. 185–188 °C, pale yellow powder; ^1^H NMR (dmso-*d*_6_) *δ* = 11.25 (d, *^3^J_NH′/2′_* = 2.5 Hz, 1H, N′-H), 10.95 (s, 1H, N-H), 8.55 (s, 1H, O-H), 8.52 (s, 1H, O′-H), 7.68 (s, 1H, 4-H), 7.56 (d, *^3^J_2′/NH′_* = 2.5 Hz, 1H, 2′-H), 7.35 (d, *^3^J_7′/6′_* = 8.7 Hz, 1H, 7′-H), 7.22 (d, *^3^J_6/7_* = 8.6 Hz, 1H, 6-H), 7.11 (d, *^3^J* (^1^H,^19^F) = 3.1 Hz, 1H, 3-H), 6.78 (dd, *^3^J_7/6_* = 8.6, *^4^J_7/9_* = 2.3 Hz, 1H, 7-H), 6.68 (dd, *^3^J_6′/7′_* = 8.7, *^4^J_6′/4′_* = 2.3 Hz, 1H, 6′-H), 6.50 (d, *^4^J_9/7_* = 2.3 Hz, 1H, 9-H), 6.33 (d, *^4^J_4′/6′_* = 2.3 Hz, 1H, 4′-H); ^13^C-NMR (dmso-*d*_6_) *δ* = 167.5 (d), 152.4, 152.3, 137.7 (d), 133.0, 130.6, 130.0, 129.9, 129.8, 128.8 (d), 124.8, 124.7, 123.8, 122.9, 112.7, 112.6, 112.5, 112.4, 110.1, 106.7, 106.5, 104.3 (d); *m/z* (ESI, %) 423.0373 (100, [C_22_H_13_ClFN_2_O_2_S]^−^ − 0.7198 ppm).

*10-(5-Cyano-1H-indol-3-yl)-2-fluoro-5H-thieno[3,2-b]carbazole-8-carbonitrile (**4c**)*. Yield 13%, mp. 160–163 °C, white yellow powder; ^1^H NMR (dmso-*d*_6_) *δ* = 12.30 (s_br_, *^3^J_NH′/2′_*, 1H, N′-H), 12.08 (s, 1H, N-H), 8.09 (d, *^3^J_2′/NH′_* = 2.5 Hz, 1H, 2′-H), 7.95 (s, 1H, 4-H), 7.81 (d, *^3^J_7′/6′_* = 8.5 Hz, 1H, 7′-H), 7.68 (dd, *^3^J_7/6_* = 8.4, *^4^J_7/9_* = 1.5 Hz, 1H, 7-H), 7.63 (d, *^3^J_6/7_* = 8.4 Hz, 1H, 6-H), 7.60 (dd, *^3^J_6′/7′_* = 8.5, *^4^J_6′/4′_* = 1.5 Hz, 1H, 6′-H), 7.47 (d, *^4^J_4′/6′_* = 1.5 Hz, 1H, 4′-H), 7.28 (d, *^3^J* (^1^H,^19^F) = 3.0 Hz, 1H, 3-H), 7.13 (d, *^4^J_9/7_* = 1.5 Hz, 1H, 9-H); ^13^C-NMR (dmso-*d*_6_) *δ* = 167.2 (d), 145.6, 141.7, 137.1 (d), 130.6, 129.2, 128.8 (d), 127.1, 125.5, 125.4, 124.8, 124.7, 123.8, 123.7, 123.6, 122.9, 118.2, 118.5, 111.8, 111.7, 110.1, 104.1, 104.6 (d), 101.5, 101.4; *m/z* (ESI, %) 405.0614 (100, [C_24_H_10_FN_4_S]^−^ − 0.5259 ppm).

*10-(6-Cyano-1H-indol-3-yl)-2-fluoro-5H-thieno[3,2-b]carbazole-7-carbonitrile (**4d**)*. Yield 24%, decomposition 183 °C, white yellow powder; ^1^H NMR (dmso-*d*_6_) *δ* = 12.27 (d, *^3^J_NH′/2′_* = 2.6 Hz, 1H, N′-H), 11.89 (s, 1H, N-H), 8.14 (d, *^3^J_2′/NH′_* = 2.6 Hz, 1H, 2′-H), 8.11 (dd, *^4^J_7′/5′_* = 1.5, *^5^J_7′/4′_* = 0.7 Hz, 1H, 7′-H), 7.93 (m, *^4^J_6/8_*, *^5^J_6/9_*, 1H, 6-H), 7.93 (s, 1H, 4-H), 7.26 (d, *^3^J* (^1^H,^19^F) = 3.2 Hz, 1H, 3-H), 7.26 (dd, *^3^J_5′/4′_* = 8.1, *^4^J_5′/7′_* = 1.5 Hz, 1H, 5′-H), 7.18 (dd, *^3^J_8/9_* = 8.2, *^4^J_8/6_* = 1.5 Hz, 1H, 8-H), 7.11 (d_br_, *^3^J_4′/5′_* = 8.1 Hz, *^5^J_4′/7′_*, 1H, 4′-H), 6.94 (d_br_, *^3^J_9/8_* = 8.2 Hz, *^5^J_9/6_*, 1H, 9-H); ^13^C-NMR (dmso-*d*_6_) *δ* = 167.3 (d), 137.7 (d), 137.0, 136.2, 134.9, 130.7, 130.6, 128.7 (d), 125.9, 125.8, 124.8, 124.7, 123.8, 122.9, 119.5, 119.4, 118.5, 118.4, 114.4, 114.3, 110.1, 104.6 (d), 99.5, 99.4; *m/z* (ESI, %) 405.0618 (100, [C_24_H_10_FN_4_S]^−^ + 0.6714 ppm).

*8-Chloro-10-(5-chloro-1H-indol-3-yl)-2-fluoro-5H-thieno[3,2-b]carbazole (**4e**)*. Yield 6%, mp. 238–241 °C, white powder; ^1^H NMR (dmso-*d*_6_) *δ* = 11.87 (s_br_, *^3^J_NH′/2′_*, 1H, N′-H), 11.58 (s, 1H, N-H), 7.88 (d, *^3^J_2′/NH′_* = 2.5 Hz, 1H, 2′-H), 7.84 (s, 1H, 4-H), 7.63 (d, *^3^J_7′/6′_* = 8.6 Hz, 1H, 7′-H), 7.47 (d, *^3^J_6/7_* = 8.6 Hz, 1H, 6-H), 7.30 (dd, *^3^J_7/6_* = 8.6, *^4^J_7/9_* = 2.1 Hz, 1H, 7-H), 7.22 (dd, *^3^J_6′/7′_* = 8.6, *^4^J_6′/4′_* = 1.9 Hz, 1H, 6′-H), 7.21 (d, *^3^J* (^1^H,^19^F) = 3.3 Hz, 1H, 3-H), 6.94 (d, *^4^J_4′/6′_* = 1.9 Hz, 1H, 4′-H), 6.85 (d, *^4^J_9/7_* = 2.1 Hz, 1H, 9-H); ^13^C-NMR (dmso-*d*_6_) *δ* = 167.5 (d), 139.4, 137.7 (d), 135.5, 130.6, 129.9, 128.8 (d), 127.8, 125.6, 125.5, 124.8, 124.7, 123.8, 122.9, 122.4, 122.3, 121.7, 121.6, 114.1, 114.0, 110.1, 104.6 (d); *m/z* (ESI, %) 422.9930 (100, [C_22_H_10_Cl_2_FN_2_S]^−^ − 0.3622 ppm).

*7-Chloro-10-(6-chloro-1H-indol-3-yl)-2-fluoro-5H-thieno[3,2-b]carbazole (**4f**)*. Yield 17%, white powder; mp. 215–218 °C, ^1^H NMR (dmso-*d*_6_) *δ* = 11.76 (d, *^3^J_NH′/2′_* = 2.2 Hz, 1H, N′-H), 11.53 (s, 1H, N-H), 7.83 (s, 1H, 4-H), 7.83 (d, *^3^J_2′/NH′_* = 2.2 Hz, 1H, 2′-H), 7.62 (“t”, *^4^J_7′/5′_*, 1H, 7′-H), 7.47 (d, *^4^J_6/8_* = 1.8 Hz, 1H, 6-H), 7.20 (d, *^3^J* (^1^H,^19^F) = 3.1 Hz, 1H, 3-H), 6.97–6.92 (m, 2H: 4′-H, 5′-H), 6.86 (d, *^3^J_9/8_* = 8.5 Hz, 1H, 9-H), 6.82 (dd, *^3^J_8/9_* = 8.5, *^4^J_8/6_* = 1.8 Hz, 1H, 8-H); ^13^C-NMR (dmso-*d*_6_) *δ* = 167.1 (d), 137.7, 137.6 (d), 136.9, 130.6, 128.8, 128.7 (d), 128.2, 128.1, 124.8, 124.3, 124.5, 123.8, 123.7, 123.6, 122.9, 121.3, 121.2, 111.9, 111.8, 110.1, 104.4 (d); *m/z* (ESI, %) 422.9933 (100, [C_22_H_10_Cl_2_FN_2_S]^−^ + 0.3091 ppm).

*8-Bromo-10-(5-bromo-1H-indol-3-yl)-2-fluoro-5H-thieno[3,2-b]carbazole (**4g**)*. Yield 5%, white powder; mp. 255–258 °C, ^1^H NMR (dmso-*d*_6_) *δ* = 11.89 (s_br_, *^3^J_NH′/2′_*, 1H, N′-H), 11.59 (s, 1H, N-H), 7.87 (d, *^3^J_2′/NH′_* = 2.6 Hz, 1H, 2′-H), 7.84 (s, 1H, 4-H), 7.58 (d, *^3^J_7′/6′_* = 8.7 Hz, 1H, 7′-H), 7.43 (d, *^3^J_6/7_* =8.5 Hz, 1H, 6-H), 7.40 (dd, *^3^J_7/6_* = 8.5, *^4^J_7/9_* = 1.7 Hz, 1H, 7-H), 7.33 (dd, *^3^J_6′/7′_* = 8.7, *^4^J_6′/4′_* = 1.9 Hz, 1H, 6′-H), 7.22 (d, *^3^J* (^1^H,^19^F) = 3.0 Hz, 1H, 3-H),7.09 (d, *^4^J_4′/6′_* = 1.9 Hz, 1H, 4′-H), 7.01 (d, *^4^J_9/7_* = 1.7 Hz, 1H, 9-H); ^13^C-NMR (dmso-*d*_6_) *δ* = 167.6 (d), 140.3, 137.5 (d), 136.4, 130.7, 130.6, 128.8, 128.6 (d), 126.0, 125.9, 124.8, 124.7, 124.6, 124.5, 123.8, 122.9, 122.5, 113.3, 113.2, 110.1, 104.6 (d); *m/z* (ESI, %) 512.8890 (100, [C_22_H_10_Br^81^BrFN_2_S]^−^ − 1.9685 ppm).

*7-Bromo-10-(6-bromo-1H-indol-3-yl)-2-fluoro-5H-thieno[3,2-b]carbazole (**4h**)*. Yield 6%, mp. 115–118 °C, pale pink powder; ^1^H NMR (dmso-*d*_6_) *δ* = 11.77 (s, *^3^J_NH′/2′_*, 1H, N′-H), 11.53 (s, 1H, N-H), 7.83 (s, 1H, 4-H), 7.81 (d, *^3^J_2′/NH′_* = 2.5 Hz, 1H, 2′-H), 7.77 (dd, *^4^J_7′/5′_* = 1.8, *^5^J_7′/4′_* = 0.6 Hz, 1H, 7′-H), 7.62 (d, *^4^J_6/8_* = 1.8 Hz, 1H, 6-H), 7.19 (d, *^3^J* (^1^H,^19^F) = 3.1 Hz, 1H, 3-H), 7.06 (dd, *^3^J_5′/4′_* = 8.5, *^4^J_5′/7′_* = 1.8 Hz, 1H, 5′-H), 6.94 (dd, *^3^J_8/9_* = 8.5, *^4^J_8/6_* = 1.8 Hz, 1H, 8-H), 6.89 (d_br_, *^3^J_4′/5′_* = 8.5 Hz, *^5^J_4′/7′_*, 1H, 4′-H), 6.80 (d, *^3^J_9/8_* = 8.5 Hz, 1H, 9-H); ^13^C-NMR (dmso-*d*_6_) *δ* = 167.3 (d), 138.5, 137.7 (d), 135.1, 130.6, 129.5, 128.8 (d), 125.7, 125.6, 125.4, 124.9, 124.8, 123.8, 123.2, 123.1, 122.9, 114.9, 114.8, 114.4, 114.3, 110.0, 104.2 (d); *m/z* (ESI, %) 512.8906 (100, [C_22_H_10_Br^81^BrFN_2_S]^−^ + 1.1408 ppm).

*2-Fluoro-10-(5-hydroxy-1H-indol-3-yl)-9H-thieno[2,3-b]carbazol-6-ol (**5a**)*. Yield 13%, mp. 187–190 °C, white powder; ^1^H NMR (dmso-*d*_6_) *δ* = 11.30 (s_br_, *^3^J_NH′/2′_*, 1H, N′-H), 10.30 (s, 1H, N-H), 8.92 (s, 1H, O-H), 8.64 (s, 1H, O′-H), 8.28 (s, 1H, 4-H), 7.64 (d, *^3^J_2′/NH′_* = 2.6 Hz, 1H, 2′-H), 7.47(d, *^4^J_5/7_* = 2.3 Hz, 1H, 5-H), 7.33 (d, *^3^J_7′/6′_* = 8.7 Hz, 1H, 7′-H), 7.25 (d, *^3^J_8/7_* = 8.6 Hz, 1H, 8-H), 7.12 (d, *^3^J* (^1^H,^19^F) = 3.0 Hz, 1H, 3-H), 6.86 (dd, *^3^J_7/8_* = 8.6, *^4^J_7/5_* = 2.3 Hz, 1H, 7-H), 6.70 (dd, *^3^J_6′/7′_* = 8.7, *^4^J_6′/4′_* = 2.4 Hz, 1H, 6′-H), 6.55 (d, *^4^J_4′/6′_* = 2.4 Hz, 1H, 4′-H); ^13^C-NMR (dmso-*d*_6_) *δ* = 167.2 (d), 152.1, 152.0, 137.6 (d), 133.9, 130.6, 130.0, 129.8, 128.6 (d), 126.4, 124.8, 124.7, 123.8, 122.9, 112.7, 112.6, 112.5, 112.4, 110.0, 106.6, 106.5, 104.2 (d); *m/z* (ESI, %) 387.0608 (51, [C_22_H_12_FN_2_O_2_S]^−^ − 0.3777 ppm); 423.0373 (100, [C_22_H_13_ClFN_2_O_2_S]^−^ − 0.5575 ppm).

*10-(5-Cyano-1H-indol-3-yl)-2-fluoro-9H-thieno[2,3-b]carbazole-6-carbonitrile (**5b**)*. Yield 3%, mp. 211–214 °C, white powder; ^1^H NMR (dmso-*d*_6_) *δ* = 12.26 (s_br_, *^3^J_NH′/2′_*, 1H, N′-H), 11.39 (s, 1H, N-H), 8.79 (s_br_, *^4^J_5/7_*, 1H, 5-H), 8.62 (s, 1H, 4-H), 8.07 (d, *^3^J_2′/NH′_* = 2.5 Hz, 1H, 2′-H), 7.75 (d, *^3^J_7′/6′_* = 8.4 Hz, 1H, 7′-H), 7.75 (dd, *^3^J_7/8_* = 8.4, *^4^J_7/5_* = 1.5 Hz, 1H, 7-H), 7.70 (s_br_, *^4^J_4′/6′_*, 1H, 4′-H), 7.58 (dd, *^3^J_6′/7′_* = 8.4, *^4^J_6′/4′_* = 1.6 Hz, 1H, 6′-H), 7.55 (d, *^3^J_8/7_* = 8.4 Hz, 1H, 8-H), 7.28 (d, *^3^J* (^1^H,^19^F) = 2.9 Hz, 1H, 3-H); ^13^C-NMR (dmso-*d*_6_) *δ* = 167.9 (d), 145.4, 141.6, 137.6 (d), 130.5, 129.1, 128.9 (d), 127.2, 125.5, 125.4, 124.8, 124.7, 123.9, 123.8, 123.5, 122.9, 118.5, 118.4, 111.7, 111.6, 110.1, 104.5 (d), 101.7, 101.6; *m/z* (ESI, %) 405.0621 (100, [C_24_H_10_FN_4_S]^−^ + 1.3950 ppm).

*6-Chloro-10-(5-chloro-1H-indol-3-yl)-2-fluoro-9H-thieno[2,3-b]carbazole (**5c**)*. Yield 5%, mp. 236–239 °C, white powder; ^1^H NMR (dmso-*d*_6_) *δ* = 11.87 (s_br_, *^3^J_NH′/2′_*, 1H, N′-H), 10.93 (s, 1H, N-H), 8.51 (s, 1H, 4-H), 8.30 (d, *^4^J_5/7_* = 2.1 Hz, 1H, 5-H), 7.89 (d, *^3^J_2′/NH′_* = 2.6 Hz, 1H, 2′-H), 7.59 (d, *^3^J_7′/6′_* = 8.6 Hz, 1H, 7′-H), 7.43 (d, *^3^J_8/7_* = 8.7 Hz, 1H, 8-H), 7.38 (dd, *^3^J_7/8_* = 8.7, *^4^J_7/5_* = 2.1 Hz, 1H, 7-H), 7.20 (d, *^3^J* (^1^H,^19^F) = 3.2 Hz, 1H, 3-H), 7.19 (dd, *^3^J_6′/7′_* = 8.6, *^4^J_6′/4′_* = 2.2 Hz, 1H, 6′-H), 7.07 (d, *^4^J_4′/6′_* = 2.2 Hz, 1H, 4′-H); ^13^C-NMR (dmso-*d*_6_) *δ* = 167.4 (d), 139.4, 137.6 (d), 137.5, 130.6, 129.9, 128.8 (d), 127.8, 125.6, 125.4, 124.7, 124.6, 123.6, 122.9, 122.4, 122.3, 121.7, 121.6, 114.1, 114.0, 110.4, 104.2 (d); *m/z* (ESI, %) 422.9935 (100, [C_22_H_10_Cl_2_FN_2_S]^−^ + 0.7885 ppm).

*6-Bromo-10-(5-bromo-1H-indol-3-yl)-2-fluoro-9H-thieno[2,3-b]carbazole (**5d**)*. Yield 6%, white powder; mp. 116–119 °C, ^1^H NMR (dmso-*d*_6_) *δ* = 11.88 (s_br_, *^3^J_NH′/2′_*, 1H, N′-H), 10.95 (s, 1H, N-H), 8.52 (s, 1H, 4-H), 8.43 (d, *^4^J_5/7_* = 2.0 Hz, 1H, 5-H) 7.88 (d, *^3^J_2′/NH′_* = 2.7 Hz, 1H, 2′-H), 7.55(d, *^3^J_7′/6′_* = 8.6 Hz, 1H, 7′-H), 7.48 (dd, *^3^J_7/8_* = 8.6, *^4^J_7/5_* = 2.0 Hz, 1H, 7-H), 7.39 (d, *^3^J_8/7_* = 8.6 Hz, 1H, 8-H), 7.30 (dd, *^3^J_6′/7′_* = 8.6, *^4^J_6′/4′_* = 2.0 Hz, 1H, 6′-H), 7.24 (d, *^4^J_4′/6′_* = 2.0 Hz, 1H, 4′-H), 7.20 (d, *^3^J* (^1^H,^19^F) = 3.0 Hz, 1H, 3-H); ^13^C-NMR (dmso-*d*_6_) *δ* = 167.6 (d), 140.2, 137.6 (d), 136.2, 130.6, 128.8, 128.4 (d), 126.0, 125.9, 124.7, 124.6, 124.5, 124.4, 123.9, 123.8, 122.9, 122.4, 122.3, 113.4, 113.2, 110.4, 104.1 (d); *m/z* (ESI, %) 512.8903 (100, [C_22_H_10_Br^81^BrFN_2_S]^−^ + 0.4065 ppm).

*2-Fluoro-4-(5-hydroxy-1H-indol-3-yl)-5H-thieno[3,2-b]carbazol-8-ol (**6a**)*. Yield 32%, mp. 193–196 °C, white powder; ^1^H NMR (dmso-*d*_6_) *δ* = 11.25 (d, *^3^J_NH′/2′_* = 2.5 Hz, 1H, N′-H), 10.27 (s, 1H, N-H), 8.91 (s, 1H, O-H), 8.62 (s, 1H, O′-H), 8.46 (s, 1H, 10-H), 7.58 (d, *^3^J_2′/NH′_* = 2.5 Hz, 1H, 2′-H), 7.46 (d, *^4^J_9/7_* = 2.4 Hz, 1H, 9-H), 7.32 (d, *^3^J_7′/6′_* = 8.7 Hz, 1H, 7′-H), 7.25 (d, *^3^J_6/7_* = 8.6 Hz, 1H, 6-H), 6.85 (dd, *^3^J_7/6_* = 8.6, *^4^J_7/9_* = 2.4 Hz, 1H, 7-H), 6.68 (dd, *^3^J_6′/7′_* = 8.7, *^4^J_6′/4′_* = 2.3 Hz, 1H,6′-H), 6.66 (dd, *^3^J* (^1^H,^19^F) = 3.0 Hz, 1H, 3-H), 6.52 (d, *^4^J_4′/6′_* = 2.3 Hz, 1H, 4′-H); ^13^C-NMR (dmso-*d*_6_) *δ* = 167.3 (d), 152.4, 152,3, 137.7 (d), 133.9, 131.8, 130.9, 129.8, 129.7 (d), 126.4, 124.9, 124.7, 122,8, 122.6, 112.7, 112.6, 112.5, 112.4, 110.1, 106.7, 106.6, 104.6 (d); *m/z* (ESI, %) 387.0603 (47, [C_22_H_12_FN_2_O_2_S]^−^ − 1.6450 ppm); 423.0368 (100, [C_22_H_13_ClFN_2_O_2_S]^−^ − 1.8039 ppm).

*4-(5-Cyano-1H-indol-3-yl)-2-fluoro-5H-thieno[3,2-b]carbazole-8-carbonitrile (**6b**)*. Yield 5%, mp. 215–218 °C, white powder; ^1^H NMR (dmso-*d*_6_) *δ* = 12.19 (s_br_, *^3^J_NH′/2′_*, 1H, N′-H), 11.31 (s, 1H, N-H), 8.78 (s, 1H, 10-H), 8.72 (d, *^4^J_9/7_* = 1.6 Hz, 1H, 9-H), 7.97 (d, *^3^J_2′/NH′_* = 2.5 Hz, 1H, 2′-H), 7.74 (dd, *^3^J_7/6_* = 8.5, *^4^J_7/9_* = 1.6 Hz, 1H, 7-H), 7.74 (d, *^3^J_7′/6′_* = 8.5 Hz, 1H, 7′-H), 7.63 (s_br_, *^4^J_4′/6′_*, 1H, 4′-H), 7.55 (dd, *^3^J_6′/7′_* = 8.5, *^4^J_6′/4′_* = 1.5 Hz, 1H, 6′-H), 7.54 (d, *^3^J_6/7_* = 8.5 Hz, 1H, 6-H), 6.77 (d, *^3^J* (^1^H,^19^F) = 2.9 Hz, 1H, 3-H); ^13^C-NMR (dmso-*d*_6_) *δ* = 167.4 (d), 145.6, 141.7, 137.7 (d), 131.8, 129.6 (d), 129.2, 127.1, 125.5, 125.4, 124.5, 124.7, 123.8, 123.7, 122.8, 122.6, 118.6, 118.6, 118.5, 111.8, 111.7, 110.1, 104.5 (d), 101.6, 101.5; *m/z* (ESI, %) 405.0611 (100, [C_24_H_10_FN_4_S]^−^ − 1.0908 ppm).

*8-Chloro-4-(5-chloro-1H-indol-3-yl)-2-fluoro-5H-thieno[3,2-b]carbazole (**6c**)*. Yield 29%, mp. 271–274 °C, white powder; ^1^H NMR (dmso-*d*_6_) *δ* = 11.80 (s_br_, 1H, N′-H), 10.86 (s, 1H, N-H), 8.68 (s, 1H, 10-H), 8.25 (d, *^4^J_9/7_* = 2.1 Hz, 1H, 9-H), 7.81 (d, *^3^J_2′/NH′_* = 2.6 Hz, 1H, 2′-H), 7.57 (d, *^3^J_7′/6′_* = 8.7 Hz, 1H, 7′-H), 7.43 (d, *^3^J_6/7_* = 8.6 Hz, 1H, 6-H), 7.36 (dd, *^3^J_7/6_* = 8.6, *^4^J_7/9_* = 2.1 Hz, 1H, 7-H), 7.19 (dd, *^3^J_6′/7′_* = 8.7, *^4^J_6′/4′_* = 2.1 Hz, 1H, 6′-H), 7.10 (d, *^4^J_4′/6′_* = 2.1 Hz, 1H, 4′-H), 6.71 (d, *^3^J* (^1^H,^19^F) = 3.2 Hz, 1H, 3-H); ^13^C-NMR (dmso-*d*_6_) *δ* = 167.3 (d), 139.4, 137.7 (d), 135.5, 131.8, 129.9, 129.7 (d), 127.8, 125.6, 125.5, 124.9, 124.7, 122.8, 122.6, 122.4, 122.3, 121.7, 121.6, 114.1, 114.0, 110.1, 104.1 (d); *m/z* (ESI, %) 422.9934 (100, [C_22_H_10_Cl_2_FN_2_S]^−^ + 0.6455 ppm).

*8-Bromo-4-(5-bromo-1H-indol-3-yl)-2-fluoro-5H-thieno[3,2-b]carbazole (**6d**)*. Yield 31%, mp. 107–110 °C, white powder; ^1^H NMR (dmso-*d*_6_) *δ* = 11.81 (s_br_, *^3^J_NH′/2′_*, 1H, N′-H), 10.88 (s, 1H, N-H), 8.69 (s, 1H, 10-H), 8.39 (d, *^4^J_9/7_* = 2.0 Hz, 1H, 9-H), 7.79 (d, *^3^J_2′/NH′_* = 2.6 Hz, 1H, 2′-H), 7.53 (d, *^3^J_7′/6′_* = 8.6 Hz, 1H, 7′-H), 7.48 (dd, *^3^J_7/6_* = 8.6, *^4^J_7/9_* = 2.0 Hz, 1H, 7-H), 7.38 (d, *^3^J_6/7_* = 8.6 Hz, 1H, 6-H), 7.30 (dd, *^3^J_6′/7′_* = 8.6, *^4^J_6′/4′_* = 1.9 Hz, 1H, 6′-H), 7.24 (d, *^4^J_4′/6′_* = 1.9 Hz, 1H, 4′-H), 6.70 (d, *^3^J* (^1^H,^19^F) = 3.2 Hz, 1H, 3-H); ^13^C-NMR (dmso-*d*_6_) *δ* = 167.4 (d), 140.3, 137.5 (d), 136.4, 131.8, 129.7 (d), 128.6, 126.0, 125.9, 124.9, 124.7, 124.6, 124.5, 123.8, 122.8, 122.6, 122.5, 122.4, 113.3, 113.2, 110.1, 104.6 (d); *m/z* (ESI, %) 512.8895 (100, [C_22_H_10_Br^81^BrFN_2_S]^−^ − 1.1627 ppm).

### 3.3. Antibacterial Activity Determination

The compounds and the used standards were dissolved in 12.5% dimethyl sulfoxide (DMSO) at concentrations of 128 µg/mL. The chosen DMSO concentration should avoid any precipitation during the following dilution procedures of the compounds, which easily dissolved in the solution. The 12.5% DMSO has been used as negative control. Further dilutions of the compounds and standard drugs in the test medium were prepared at the required quantities of 64, 32, 16, 8, 4, 2, 1, 0.5 and 0.25 µg/mL concentrations with Mueller-Hinton broth, containing beef infusion solids (20 g/L), casein hydrolysate (17.5 g/L) and starch (1.5 g/L). The minimum inhibitory concentrations (MIC) were determined using the two-fold serial dilution technique. All the compounds were tested for their in vitro growth inhibitory activity against MRSA USA300 LAC * lux with a bioluminescence gene [23], and in case of an activity < 16 µg/mL the respective compounds were tested against MRSA JE2 as a variant of USA300 LAC * with cured resistance genes located on the plasmids [24], against MSSA ATCC6538 and against the MSSA HG003. The cultures were obtained from Mueller–Hinton broth (Difco) for all the bacterial strains after 24 h of incubation at 37 ± 1 °C. Testing was carried out in Mueller–Hinton broth at pH 7.4. The final inoculum size was 5 × 10^5^ CFU/mL for the antibacterial assay. A set of tubes containing only inoculated broth was used as control. After incubation for 24 h at 37 ± 1 °C, the last tube with no visible microorganism growth was recorded to represent the MIC (expressed in µg/mL). Under similar conditions, no activities were observed against Gram-negative *E. coli*.

### 3.4. Overexpression of His-Tagged MRSA Pyruvate Kinase

MRSA PK was PCR-amplified from *S. aureus* USA300 LAC *, cloned into the expression vector pET-28a (Novagen, Merck, Darmstadt, Germany) and transformed to strain *E. coli* BL21 (D3). The resulting strain *E. coli* D3 pET28a LAC *pyk was grown overnight in 10 mL of LB-medium containing 50 µg/mL kanamycin, then diluted 1:100 in 250 mL LB-Medium with 50 µg/mL kanamycin and incubated at 37 °C until an OD600 of approximately 0.6. Overexpression of PK was induced by adding a final concentration of 1 mM sterile isopropyl-β-D-thiogalactopyranosid (IPTG) followed by growth for 24 h. The cell suspension was centrifuged at 4000 rpm and 4 °C for 10 min and the pellet was suspended in 10 mL of PBS. After centrifugation for another 10 min at 4000 rpm and 4 °C the pellet was stored at −20 °C until further use.

### 3.5. Purification of His-Tagged Proteins

The overexpressed His-tagged PK was purified using the Protino^®^ Ni-TED 2000 Kit from Macherey Nagel (Düren, Germany). Briefly, the bacterial pellet containing the overexpressed PK was thawed on ice, suspended in 5 mL LEW buffer and 5 µL DNAse I (5 mg/mL), 100 µL protease inhibitor cocktail and 500 µL lysozyme (10 mg/mL) were added to lyse the bacteria and bacterial DNA. The sample was incubated for 30 min at 4 °C while rolling at 60 rpm. For later SDS-page analysis, a sample of 80 µL of the crude lysate was taken, mixed with 20 µL SDS sample buffer and incubated for 10 min at 95 °C. The remaining lysate was centrifuged at 10,000 rpm and 4 °C for at least 40 min until the supernatant was clear. The supernatant was then filtered with a 0.2 µm filter. The column was calibrated twice, using 2 mL of LEW buffer, and the lysate was put onto the column. The flowthrough was collected and an SDS sample was prepared. The column was washed twice with 5 mL of LEW buffer and the flowthrough was collected again for SDS page analysis. To elute the protein, LEW buffer containing different amounts of imidazole (10 mM, 50 mM, 125 mM and 250 mM) were put on the column, and each fraction was collected in a separate 15 mL tube. From each fraction, an SDS sample was taken for later analysis and the protein fractions were stored at 4 °C.

### 3.6. Pyruvate Kinase Assay

The Kinase Glo Assay (Promega, Walldorf, Germany) was used to measure the PK activity. It is based on the detection of ATP, which is generated by PK, and is detected by measurement of luminescence. First, the purified PK was adjusted to a final concentration of 300 nM in LEW buffer. The samples were prepared in 1.5 mL micro tubes by adding the intended amount of PK and compounds or DMSO to D_2_O to reach a total volume of 60 µL per tube. Samples lacking enzyme and compound, as well as PK without compound, served as negative and positive controls. The final concentration of the pyruvate kinase was 15 nM. The compounds were tested in concentrations of 0.5 µM, 5 µM, 10 µM, 20 µM and 40 µM. The reaction was initiated by adding 140 µL of master mix containing 60 mM Na^+^ – HEPES, 67 mM KCl, 2 mM ADP, 10 mM PEP and 6.7 mM MgCl_2_ to each tube, vortexing briefly, and incubating in a 30 °C heating block for 5 min. Thereafter, the reaction was terminated by transferring the tubes into a 95 °C heating block and leaving them to denaturate for 1 min. Finally, the tubes were kept on ice and centrifuged briefly to collect the condensate. Then, 50 µL of each sample were transferred into the wells of a white 96-well-plate. 50 µL of Kinase-Glo were added to each well before incubating the plate in the dark at room temperature for 10 min. Ultimately, the luminescence was measured in a TECAN plate reader. In order to be able to quantify the amount of ATP generated by PK, an ATP dilution series ranging from 400 µM to 1.5625 µM plus a sample containing 0 µM of ATP were included. The amount of ATP generated by the kinases was calculated using GraphPad Prism 5 with help of the ATP standard curve.

## 4. Conclusions

The ever-increasing bacterial resistance to established and also new antibiotics urgently requires the development of novel antibacterial compounds that could address novel target structures. We developed accessible novel antibacterial compounds in a simple one-pot synthesis, which can be purified in a one-step procedure. Within our compound classes, we identified novel lead compounds with high antibacterial activities in nanomolar range for indolobenzothiophenes with cyano indole substitutions. Hydroxyl and chloro indole substitutions resulted in low micromolar activities. Highly antimicrobial substances efficiently inhibited the MRSA pyruvate kinase, which is probably the molecular target of the novel compounds. The results presented here encourage further in vivo studies to combat antibacterial drug resistance in one of the most important bacterial pathogens.

## Figures and Tables

**Table 1:** MRSA and MSSA inhibition data of target compound classes **3**–**6** expressed as MIC values.

MIC [µg/mL] ^[a]^					
MRSA			MSSA		
Cpd.	R^1^	Lac * lux	JE2	HG003 ATCC6538	
**3a**	H	1	2	3	2
**3b**	5-OH	8	8	8	6
**3c**	6-OH	2	2	2	3
**3d**	5-CN	0.75	0.75	2	1
**3e**	6-CN	1	1	2	1.5
**3f**	5-Cl	2	2	3	4
**3g**	6-Cl	1	1	4	3
**3h**	5-Br	2	2	8	4
**3i**	6-Br	4	3	6	4
**4a**	H	2	2	3	2
**4b**	5-OH	6	6	8	8
**4c**	5-CN	4	24	12	3
**4d**	6-CN	1.5	1	3	0.75
**4e**	5-Cl	2	2	6	8
**4f**	6-Cl	2	2	3	3
**4g**	5-Br	3	4	4	2
**4h**	6-Br	3	2	4	8
**5a**	5-OH	4	3	3	2
**5b**	5-CN	64	n.d. ^[b]^	n.d. ^[b]^	n.d. ^[b]^
**5c**	5-Cl	4	3	6	3
**5d**	5-Br	8	8	24	6
**6a**	5-OH	4	4	8	6
**6b**	5-CN	32	n.d. ^[b]^	n.d. ^[b]^	n.d. ^[b]^
**6c**	5-Cl	4	4	8	8
**6d**	5-Br	8	8	16	8
oxacillin		24	24	0.5	1
ciprofloxacin		32	32	1	2

^[a]^ Mean of two experiments. ^[b]^ n.d. not determined.

## Data Availability

Data is contained within the article and supplementary material.

## Supplementary Materials

The following supporting information can be downloaded at: https://www.mdpi.com/article/10.3390/ph15091138/s1.
