# Supplementary material for: Novel Effective Fluorinated Benzothiophene-Indole Hybrid Antibacterials against S. aureus and MRSA Strains
Source: Pharmaceuticals (Basel). 2022 Sep 13;15(9):1138. doi: 10.3390/ph15091138 (PMC9502754; doi:10.3390/ph15091138)
Supplement: Supplementary file 1 [file pharmaceuticals-15-01138-s001.zip › pharmaceuticals-1819316-supplementary.pdf]

Supporting Information

Communication

Novel Effective Fluorinated Benzothiophene-Indole Hybrid Antibacterials against *S. aureus* and MRSA Strains

Marius Seethaler [1], Tobias Hertlein [2], Elisa Hopke [2], Paul Köhling [2], Knut Ohlsen [2], Michael Lalk [3] and Andreas Hilgeroth [1,*]

[1] Institute of Pharmacy, Martin Luther University Halle-Wittenberg, 06120 Halle, Germany
[2] Institute of Molecular Infection Biology, Julius Maximilians University Würzburg, 97080 Würzburg, Germany
[3] Institute of Biochemistry, Ernst Moritz Arndt University Greifswald, 17489 Greifswald, Germany
* Correspondence: andreas.hilgeroth@pharmazie.uni-halle.de; Tel.: +49-345-55-25168

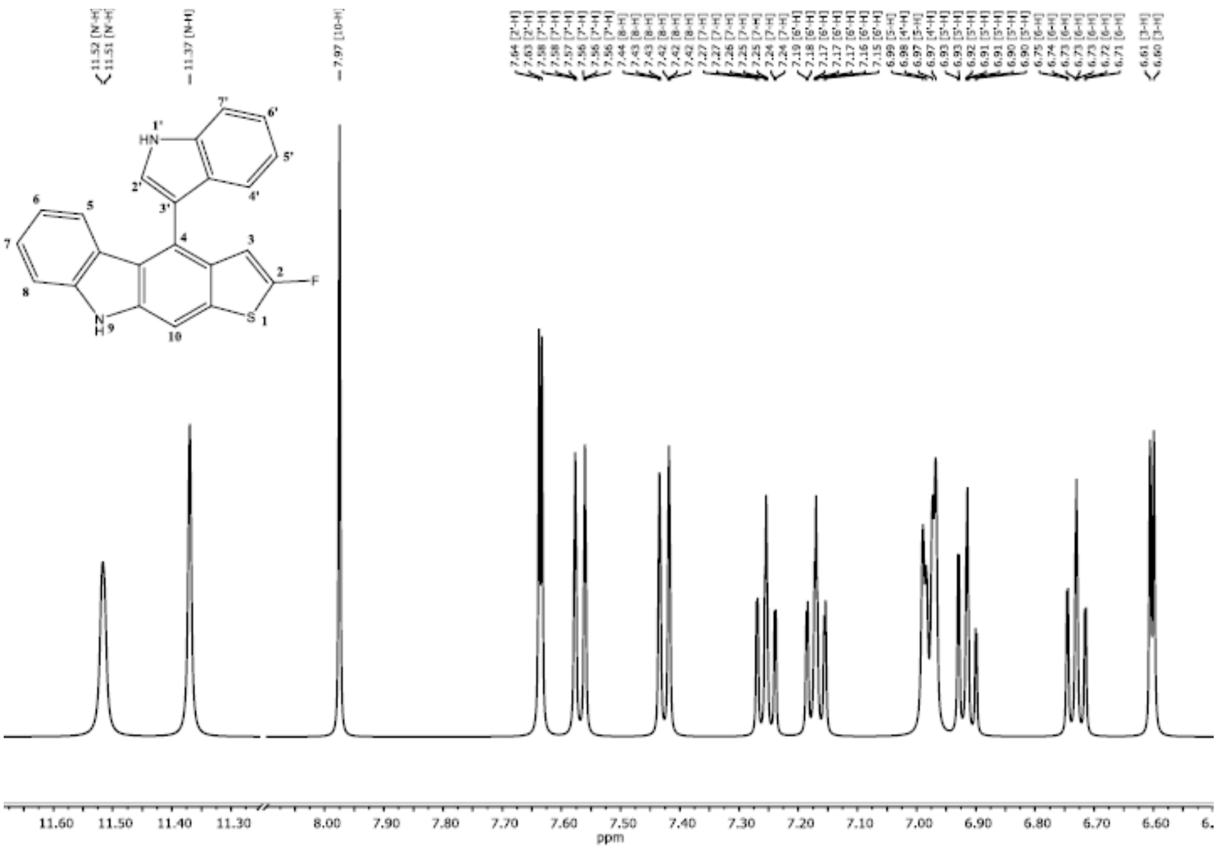

^{1}H NMR spectrum of compound **3a**

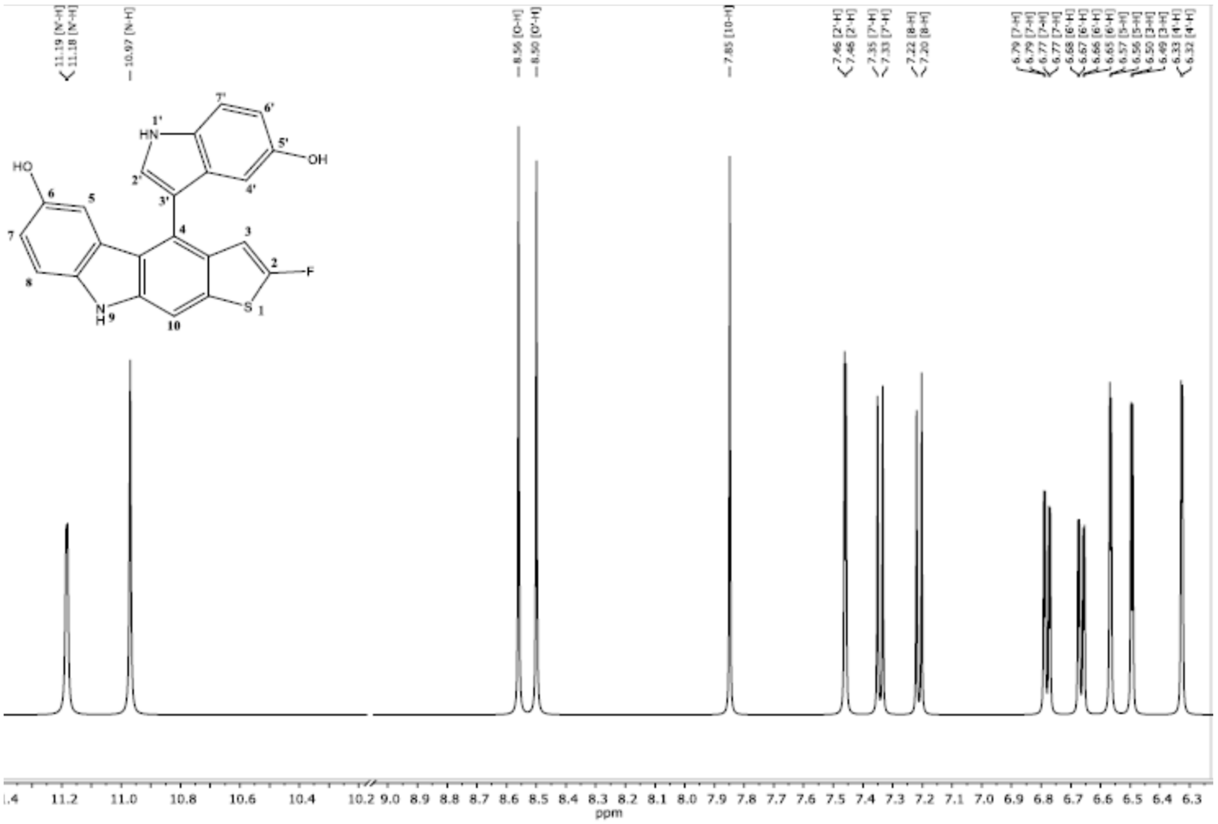

^{1}H NMR spectrum of compound **3b**

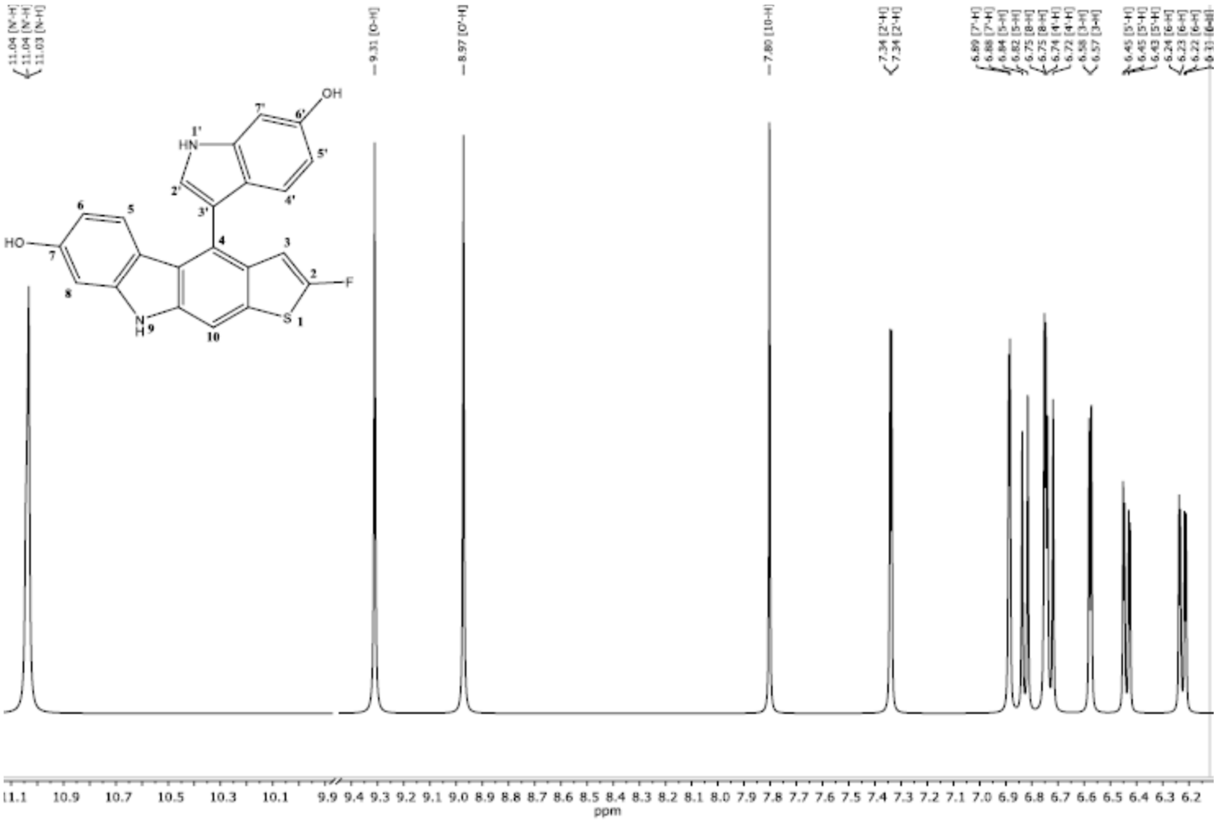

^{1}H NMR spectrum of **3c**

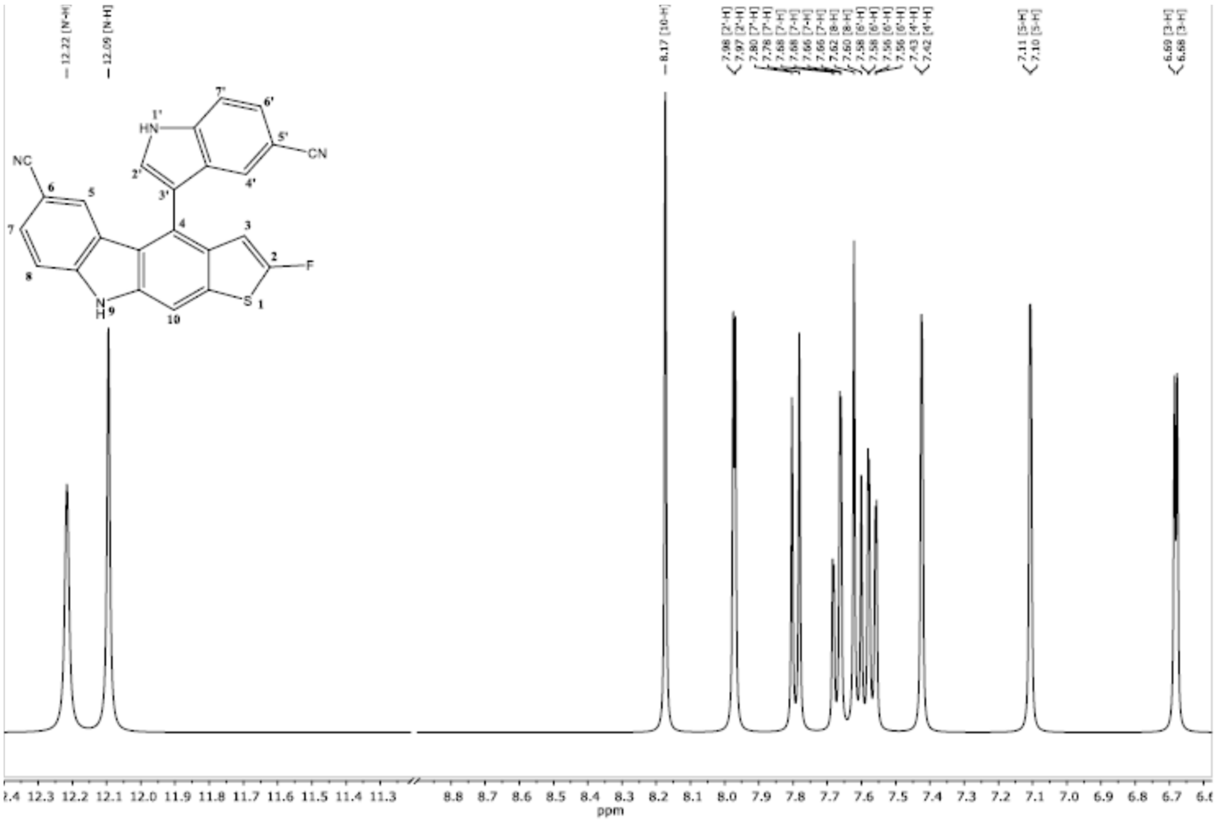

^{1}H NMR spectrum of **3d**

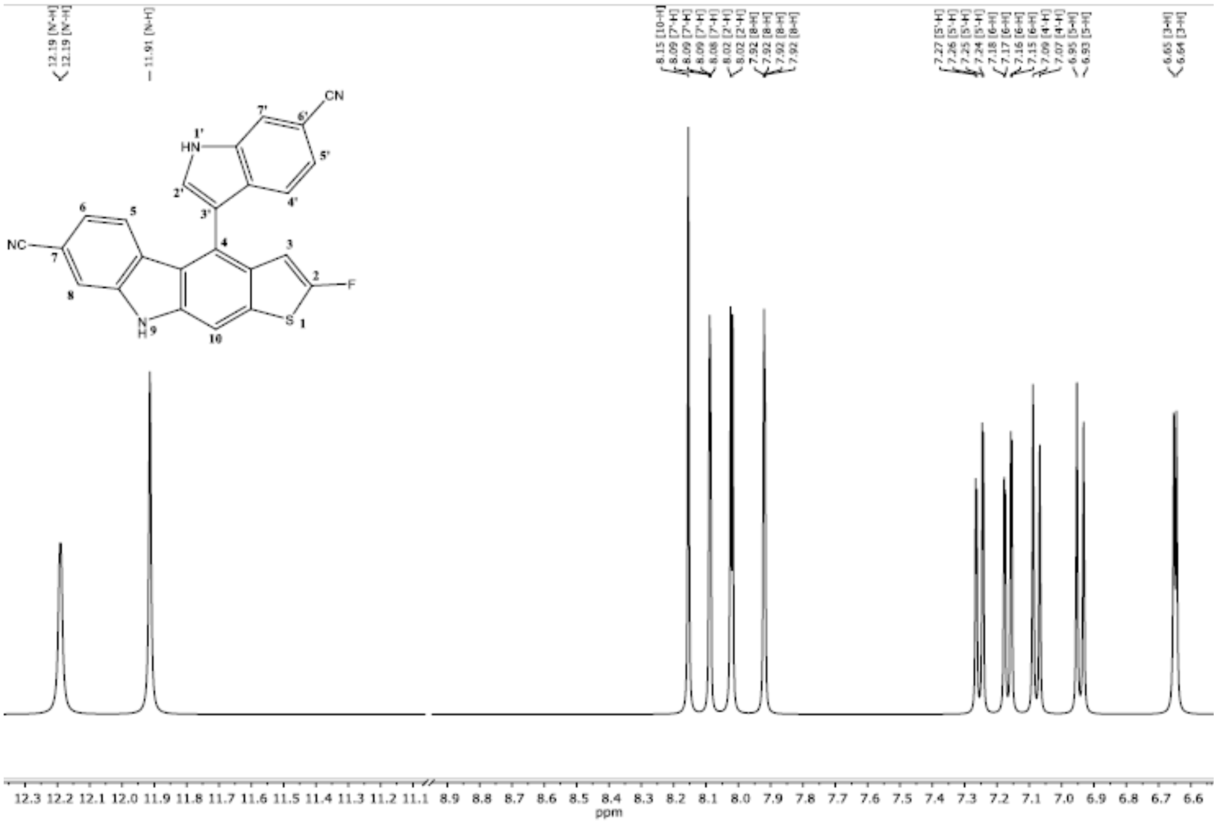

^{1}H NMR spectrum of **3e**

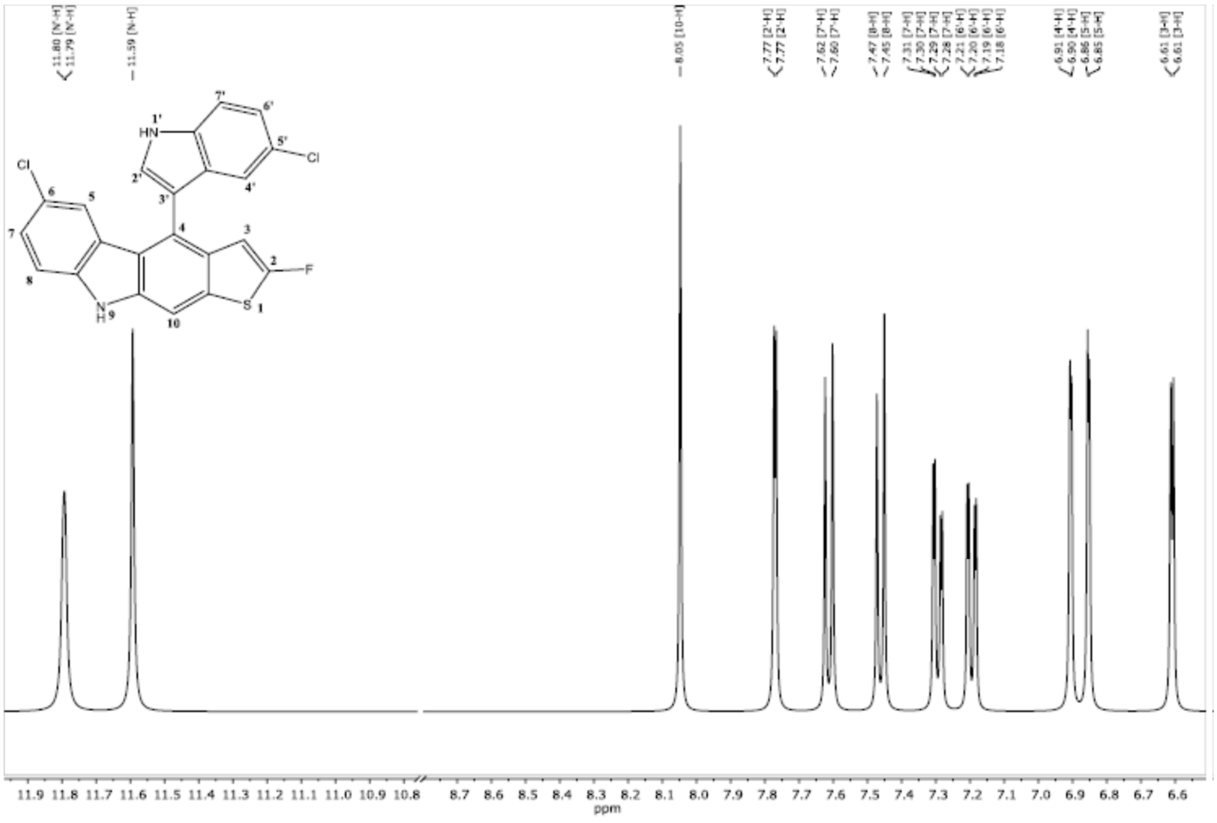

^{1}H NMR spectrum of **3f**

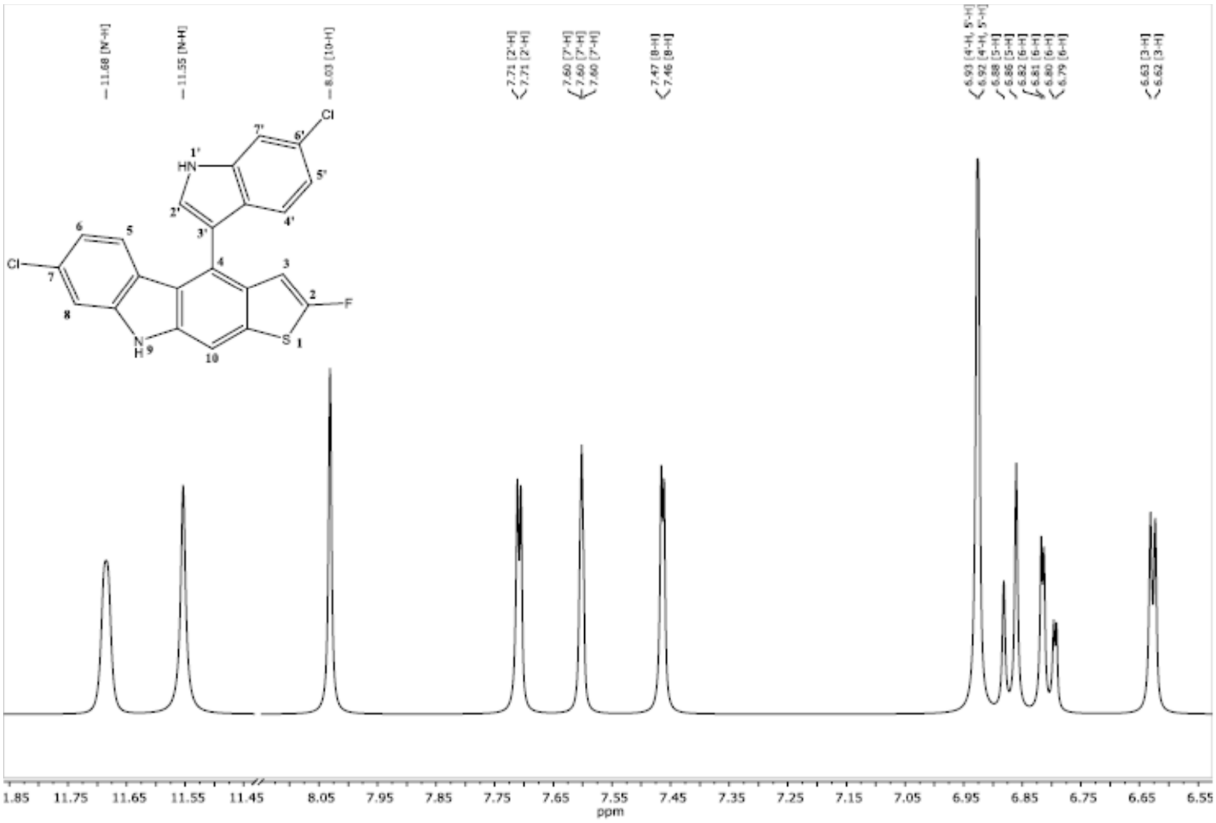

^{1}H NMR spectrum of **3g**

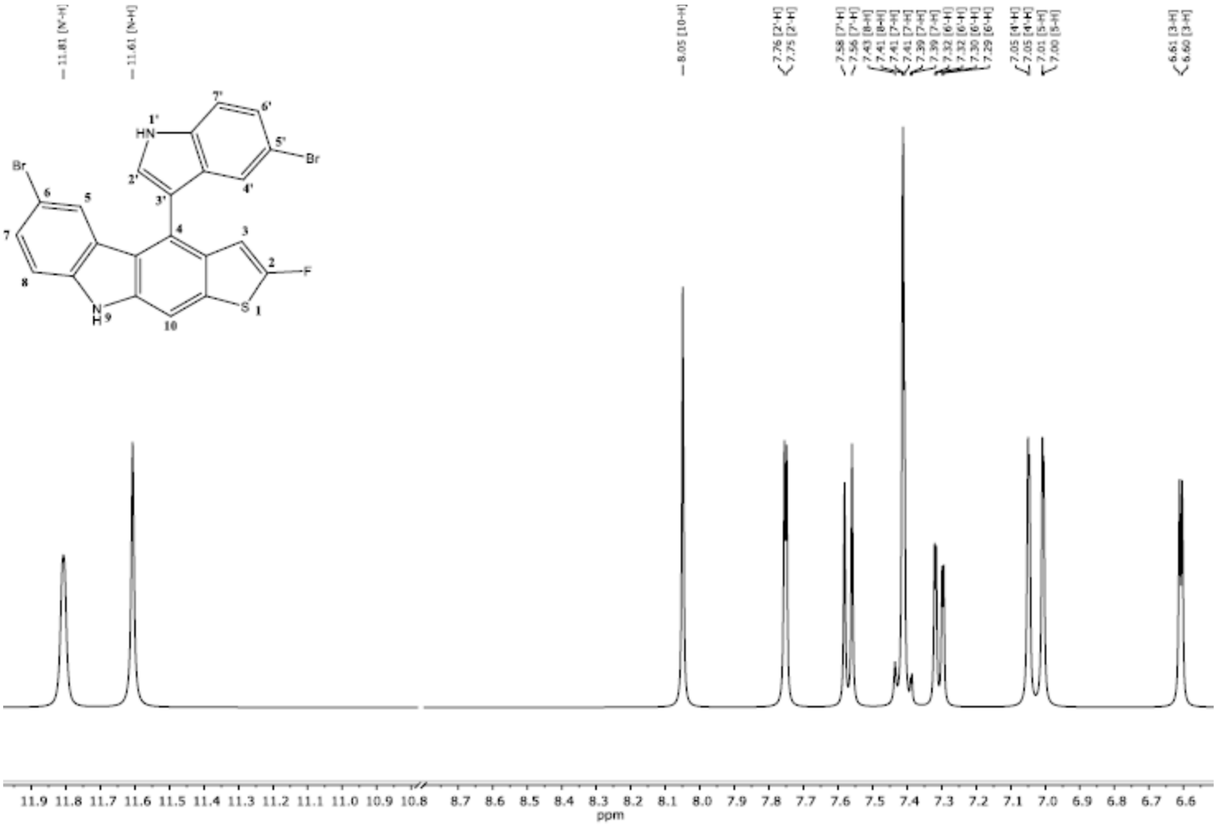

^{1}H NMR spectrum of **3h**

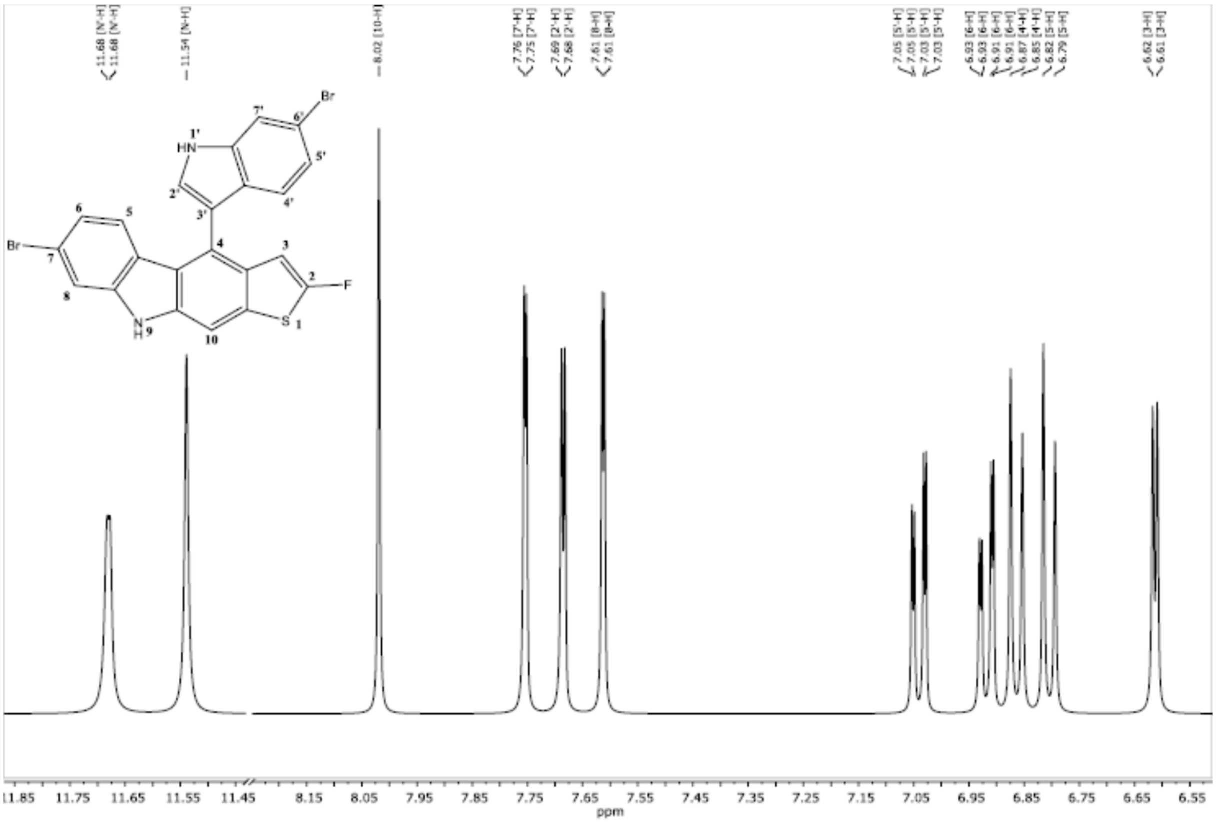

^{1}H NNR spectrum of **3i**

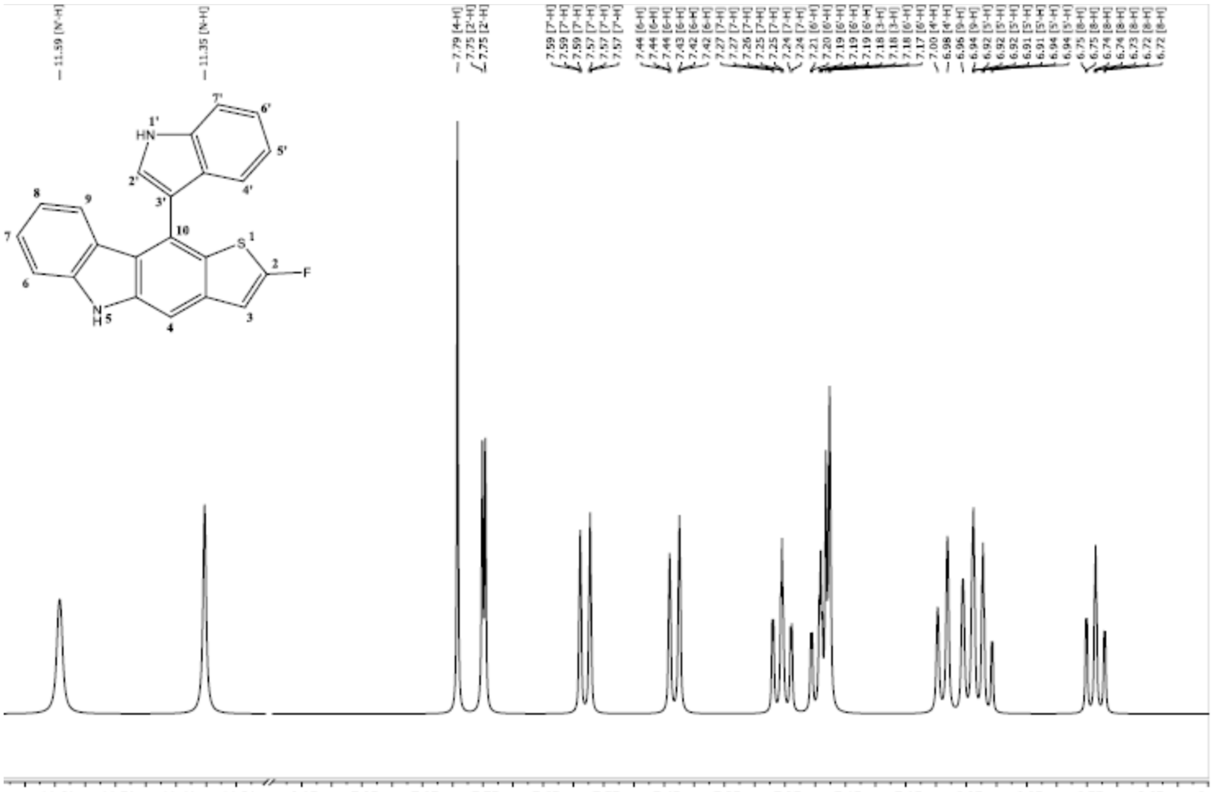

^{1}H NMR spectrum of **4a**

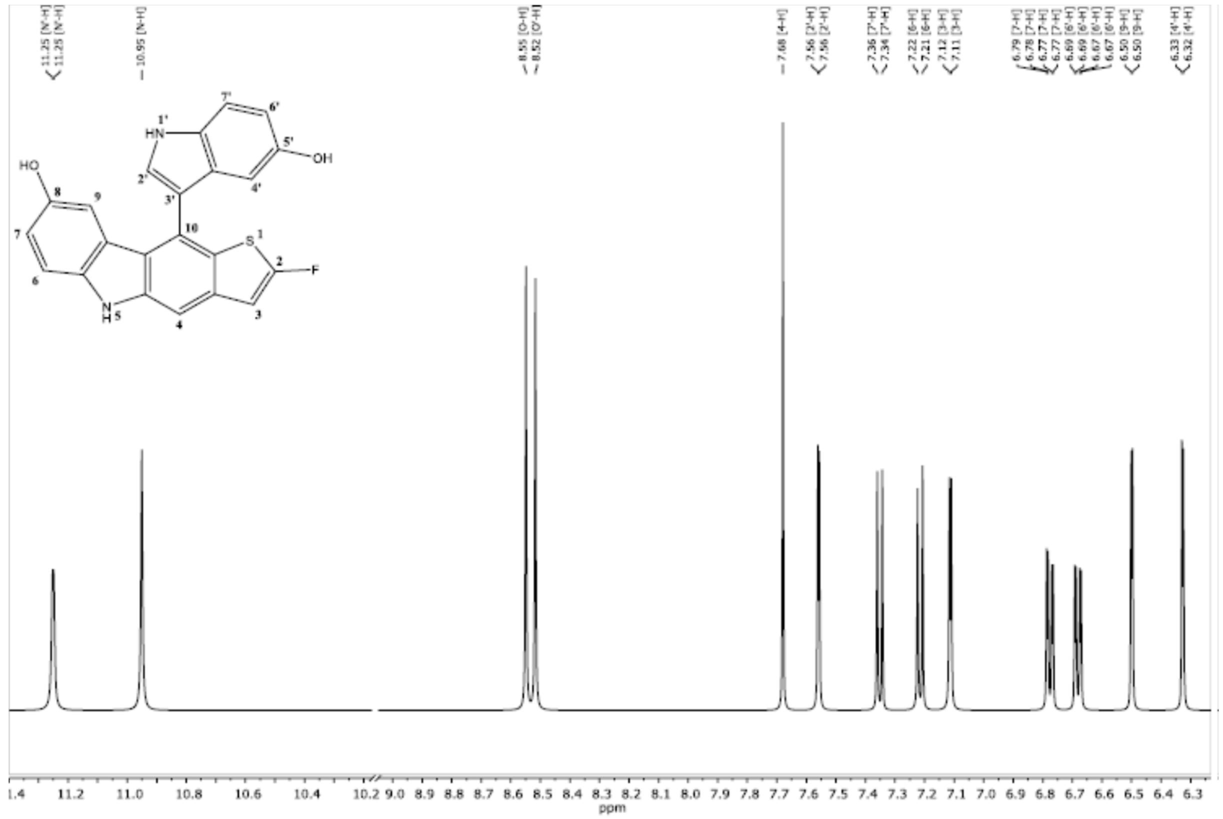

^{1}H NMR spectrum of **4b**

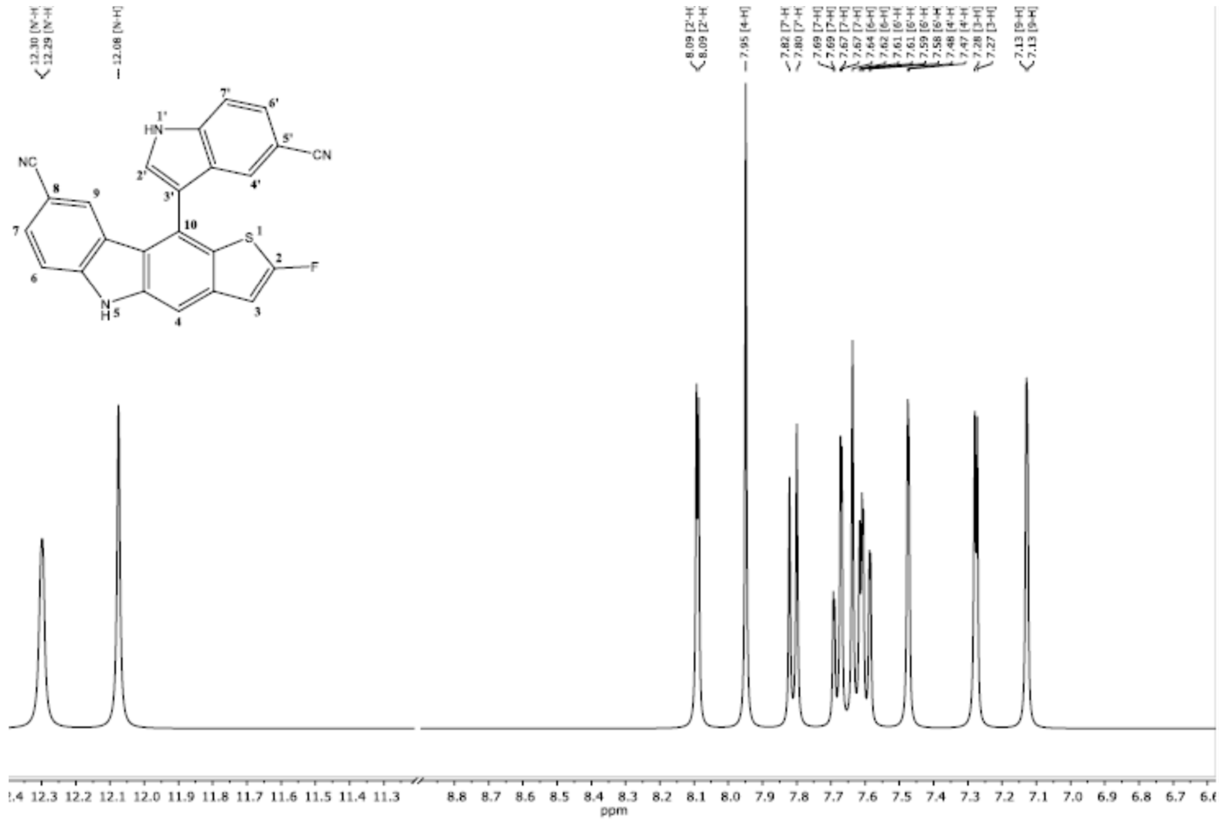

^{1}H NMR spectrum of **4c**

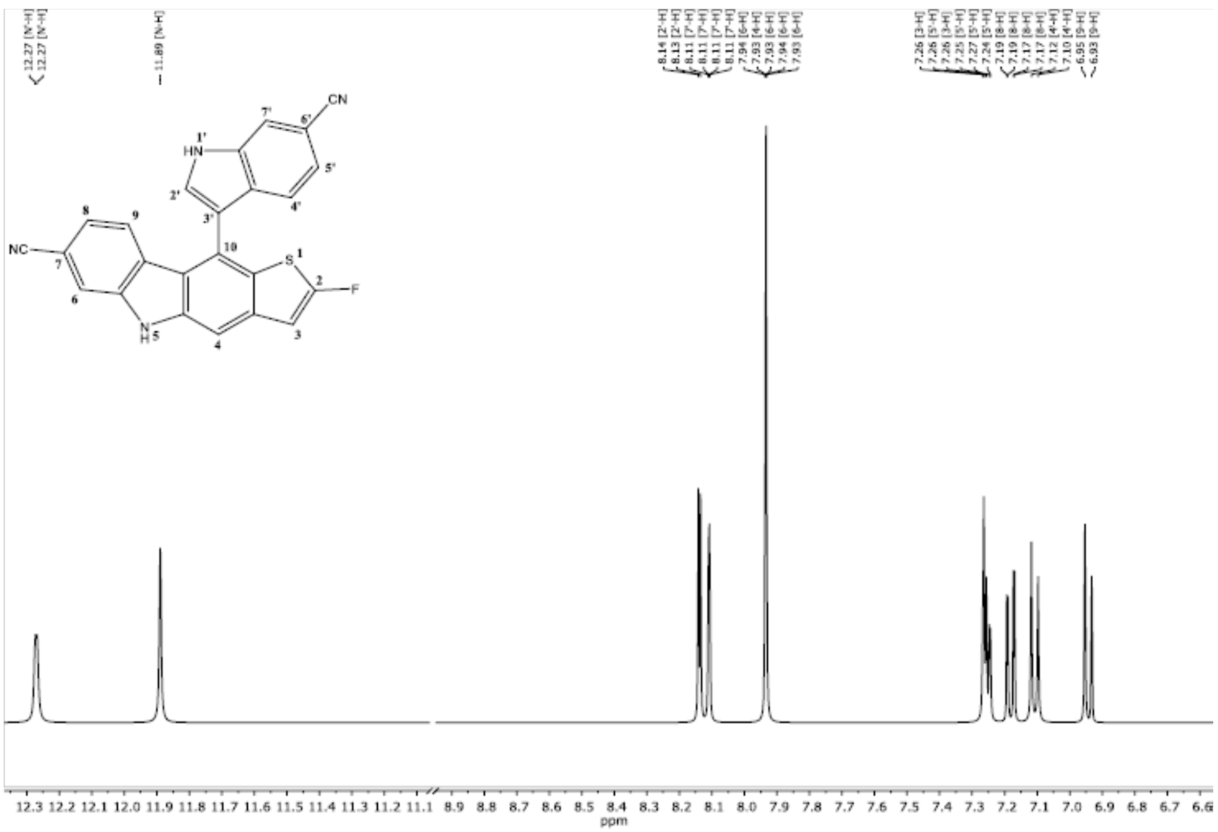

¹H NMR spectrum of **4d**

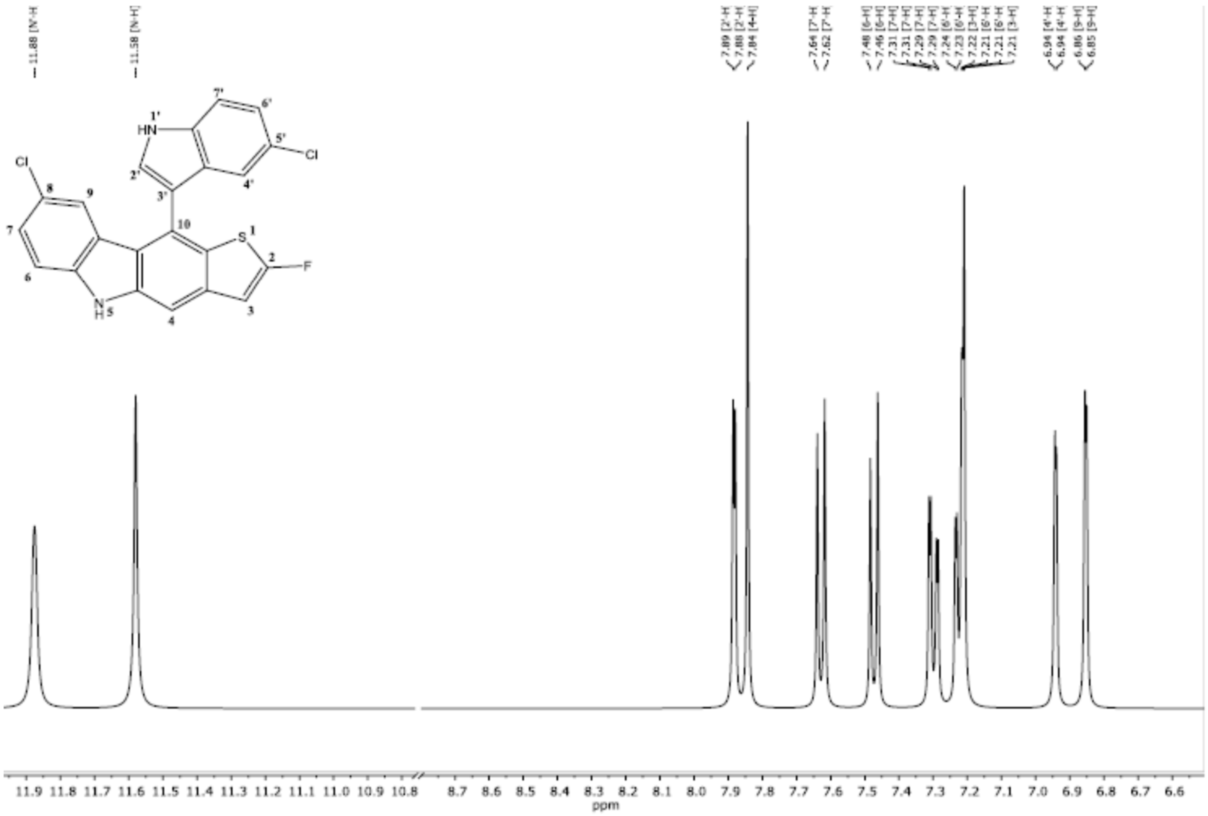

¹H NMR spectrum of **4e**

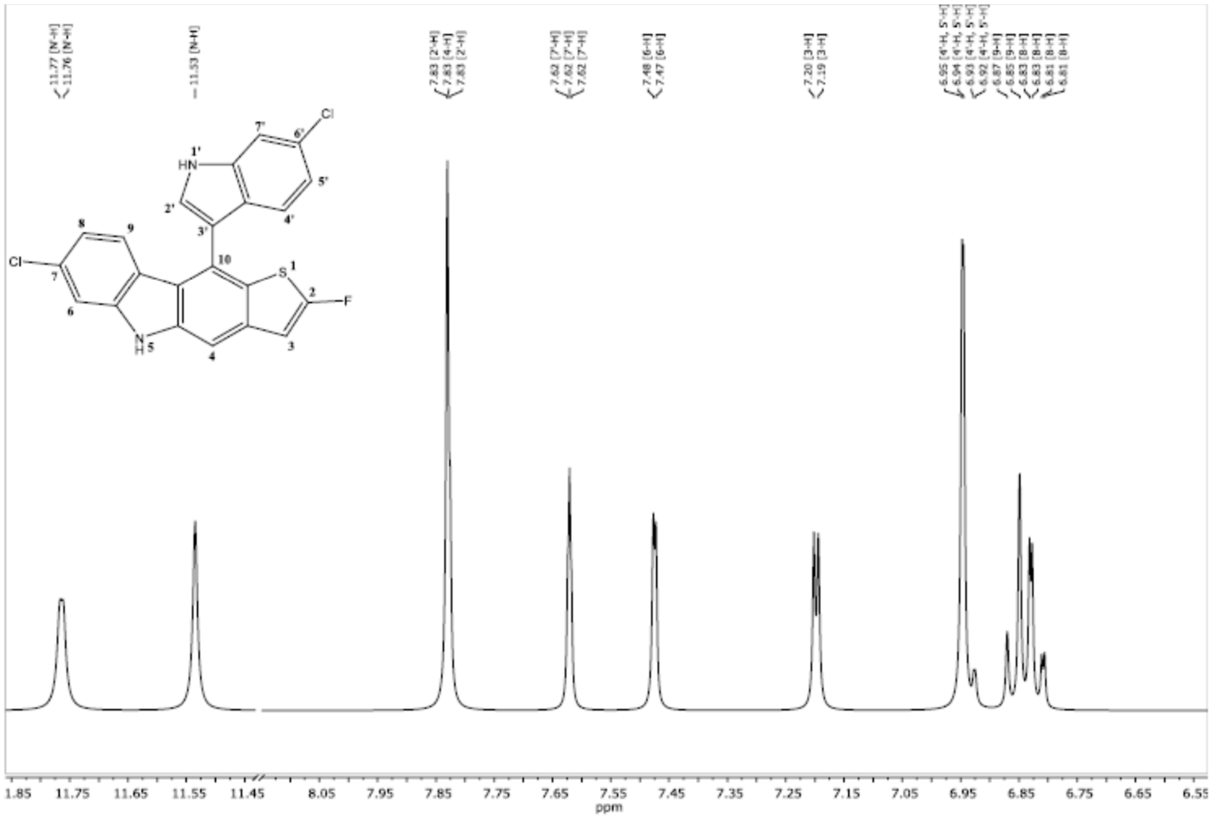

^{1}H NMR spectrum of **4f**

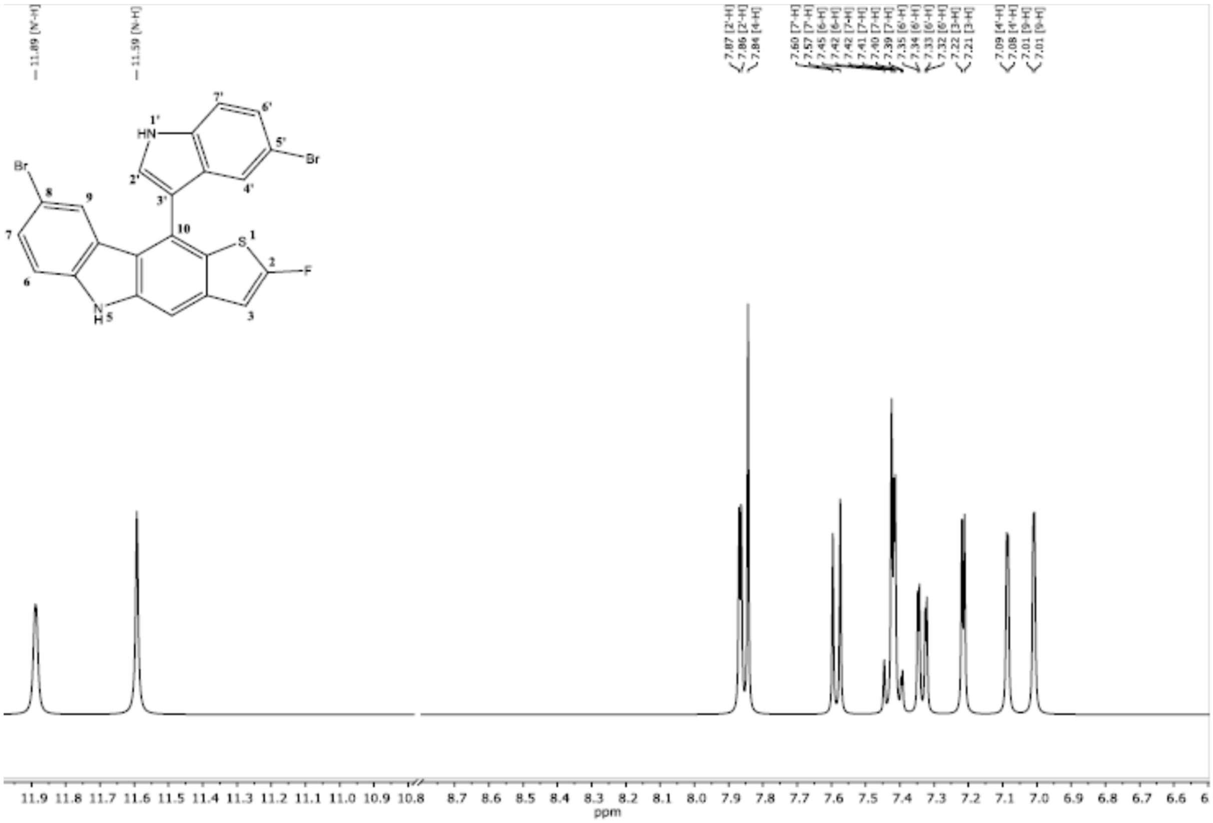

^{1}H NMR spectrum of **4g**

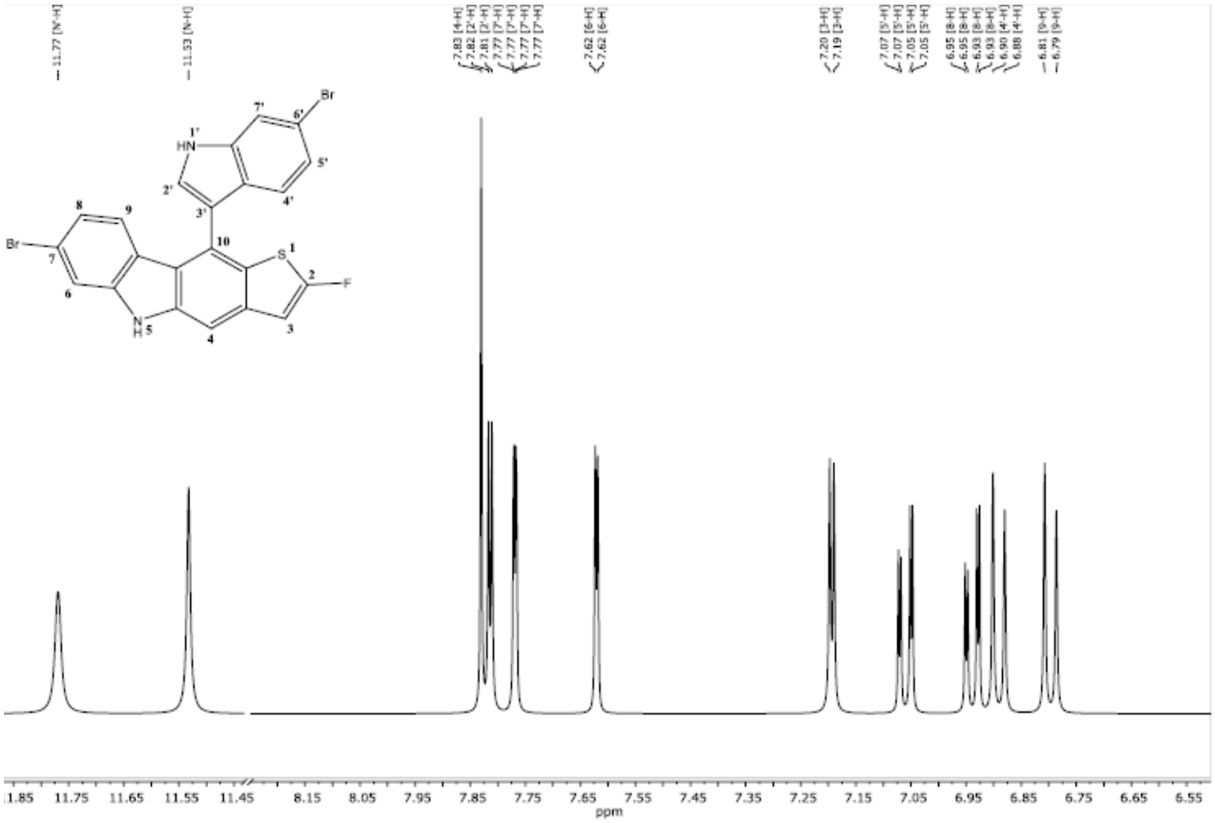

^{1}HNMR spectrum of **4h**

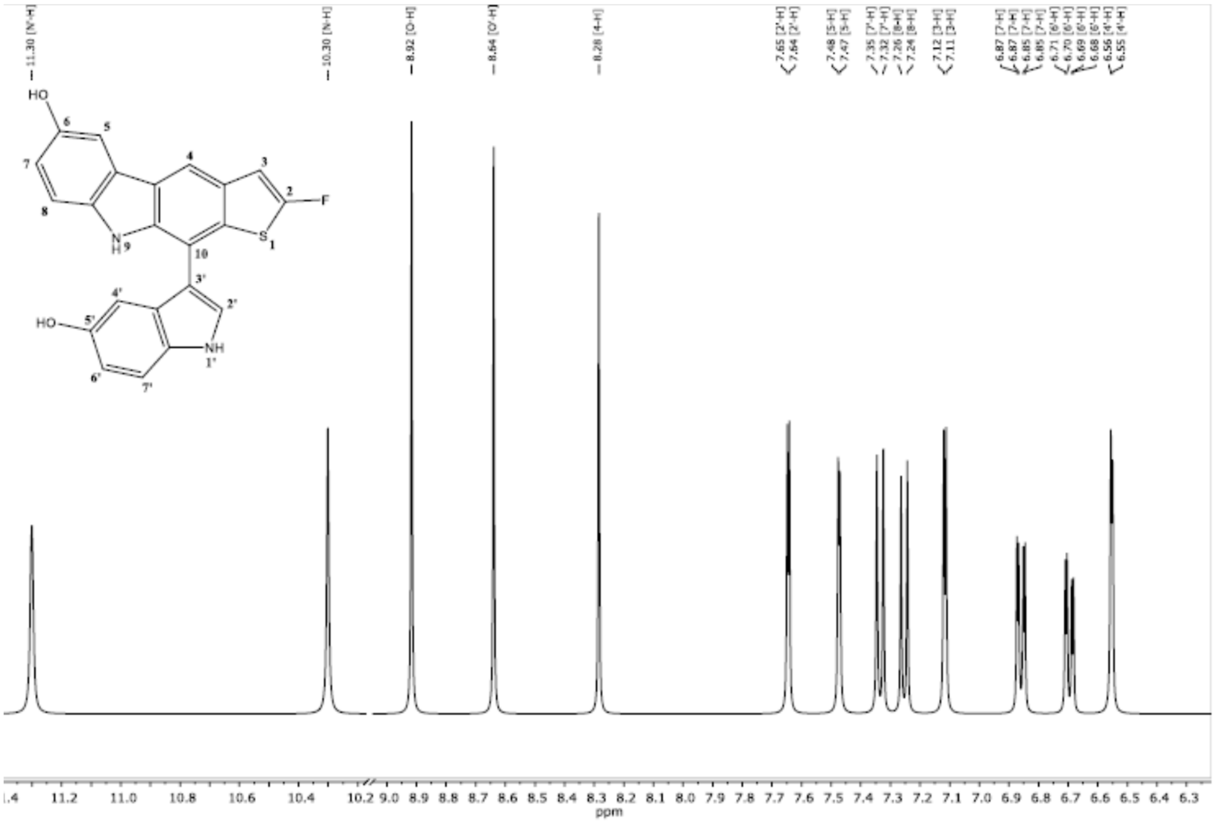

^{1}H NMR spectrum of **5a**

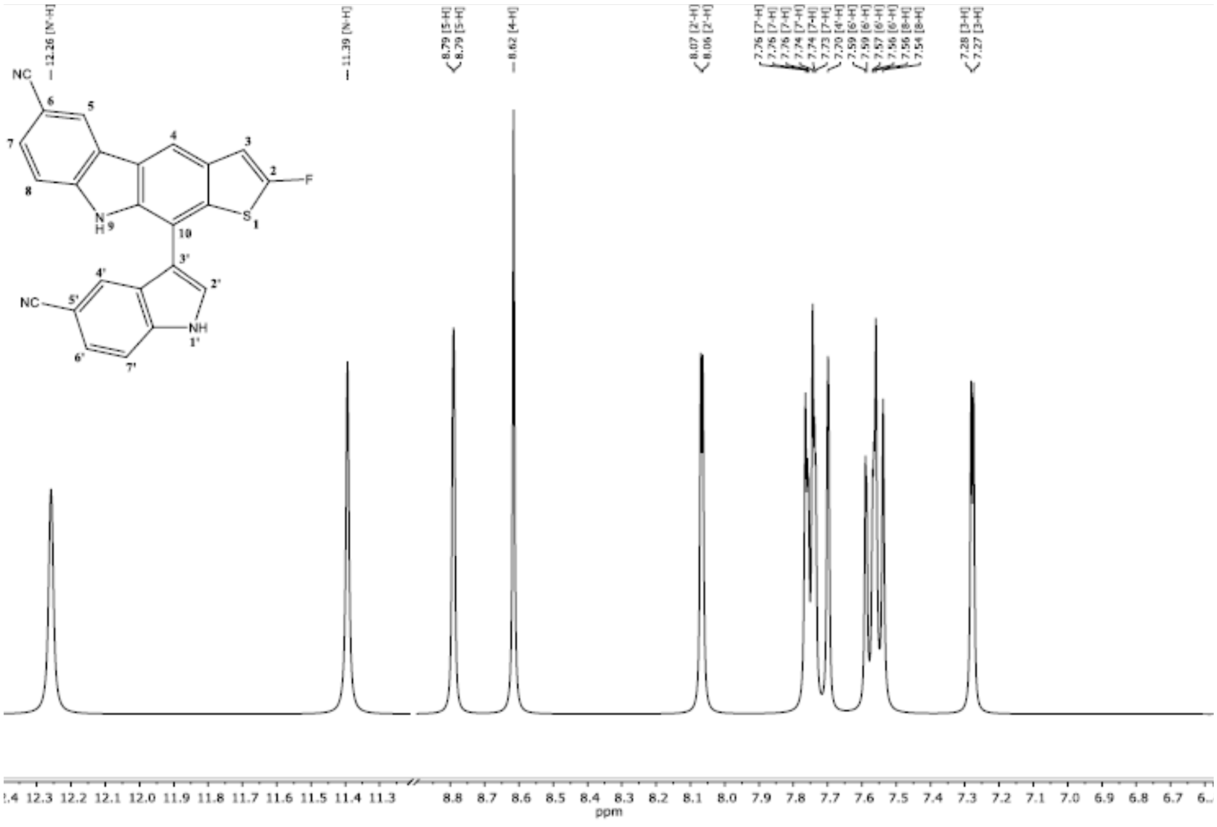

¹H NMR spectrum of **5b**

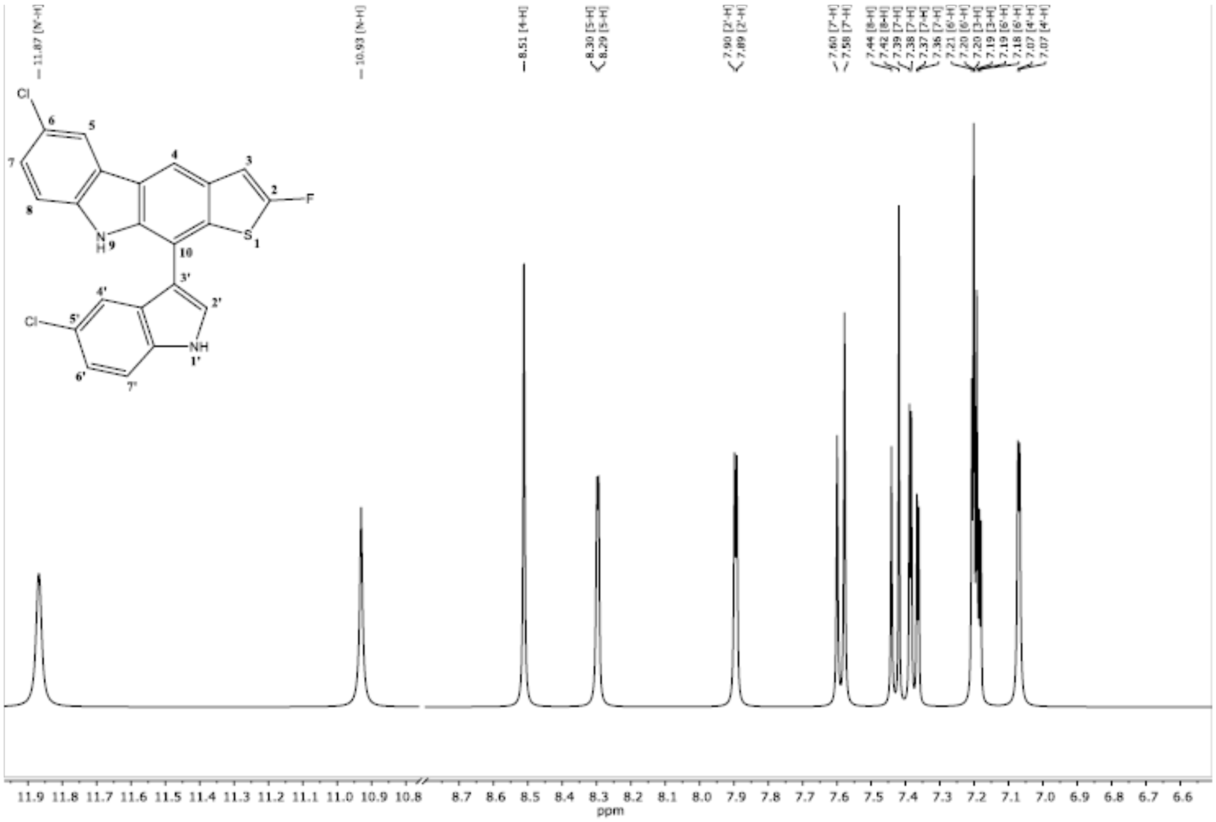

¹H NMR spectrum of **5c**

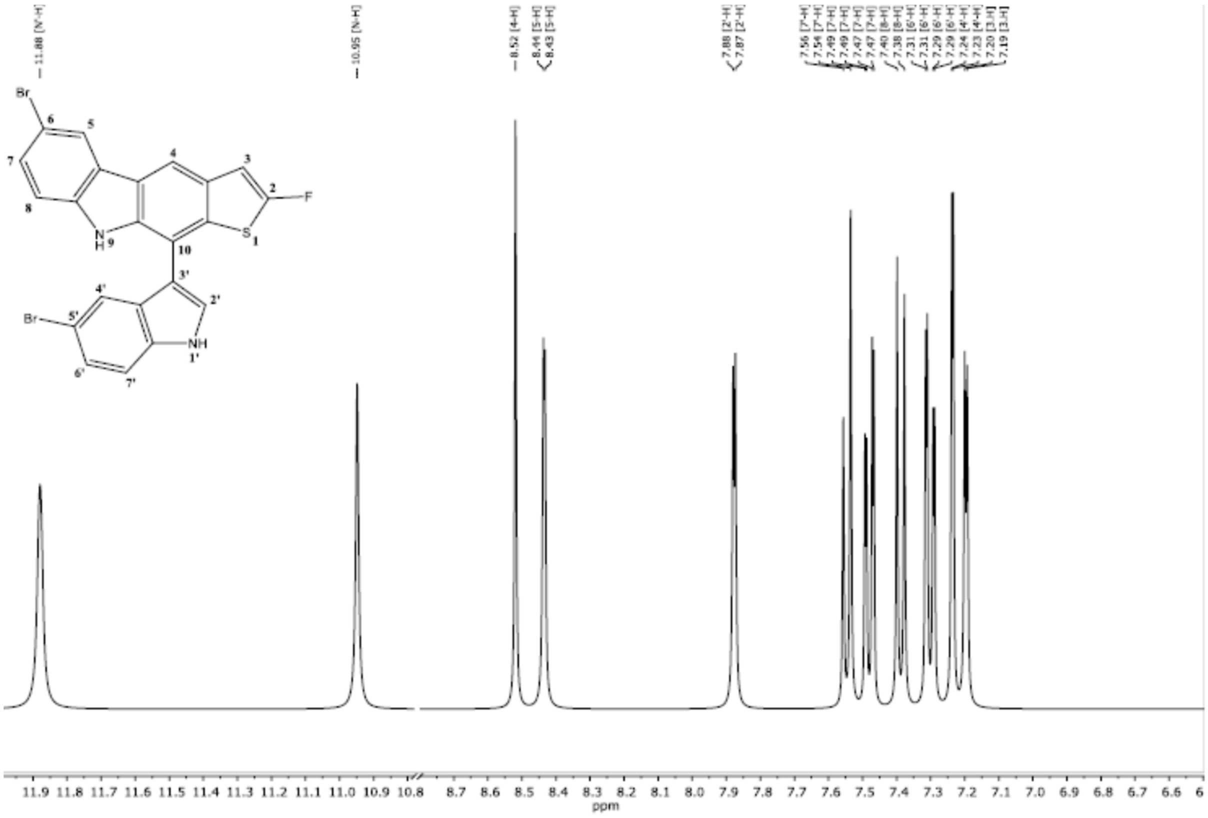

^{1}H NMR spectrum of **5d**

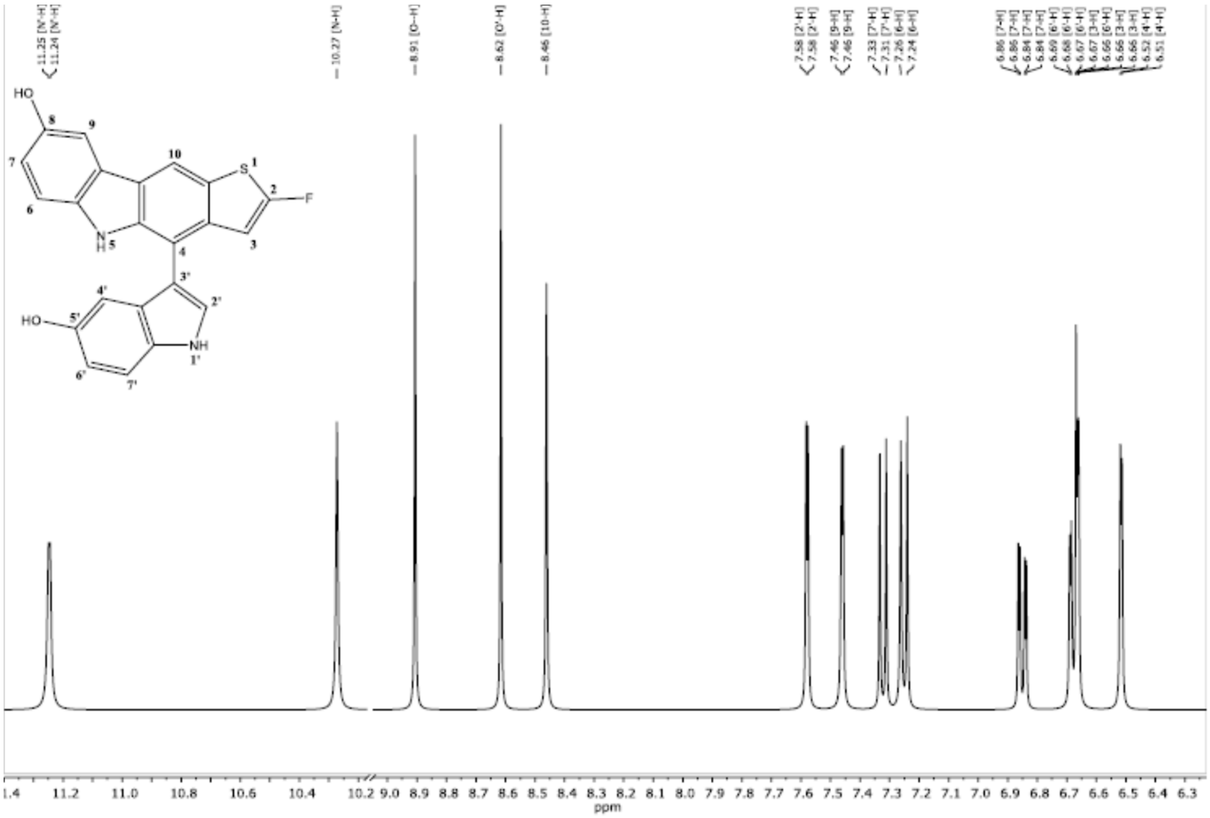

^{1}H NMR spectrum of **6a**

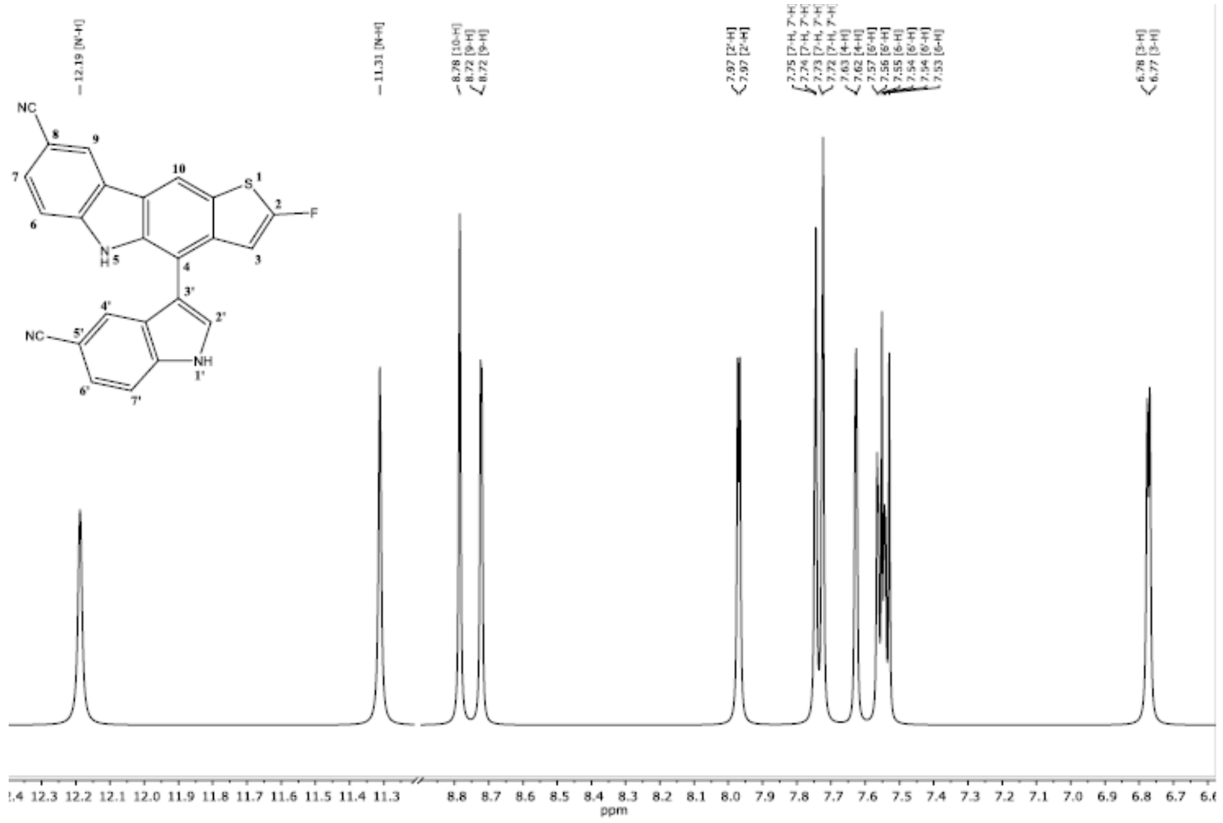

^{1}H NMR spectrum of **6b**

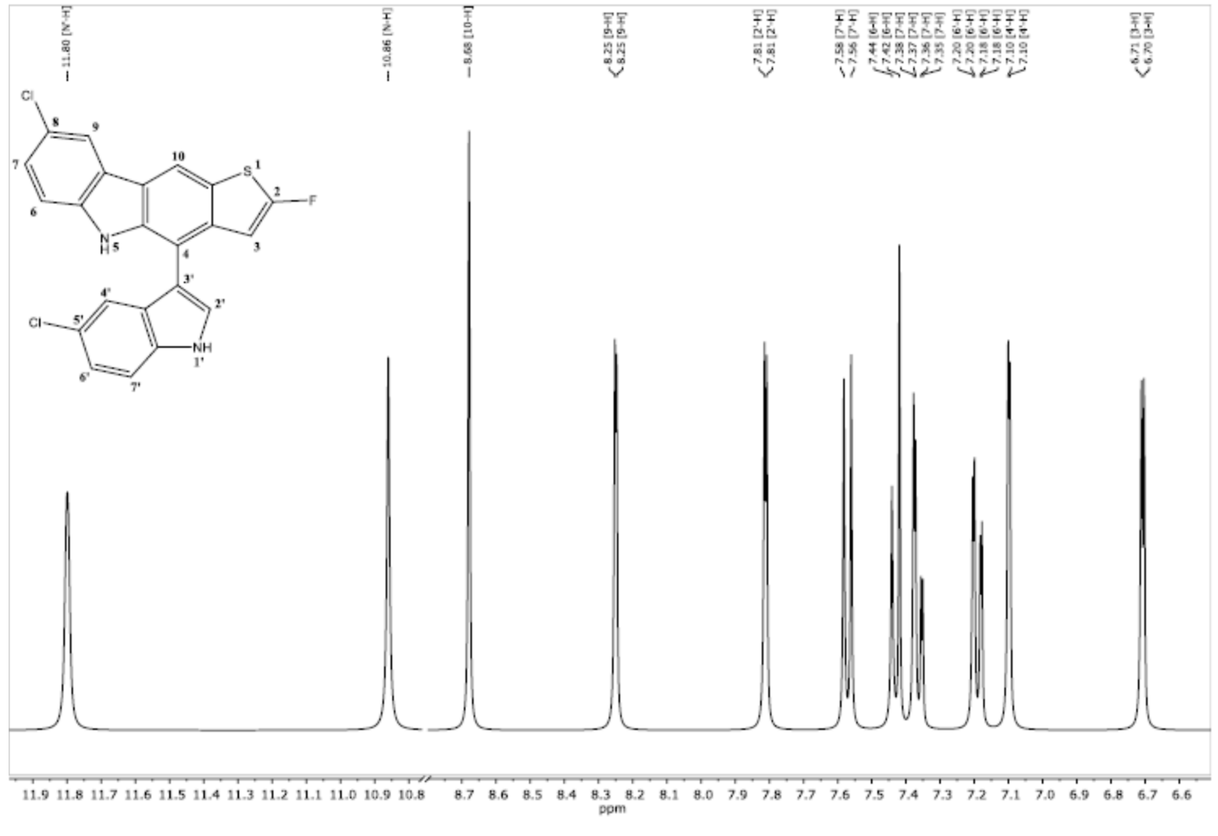

^{1}H NMR spectrum of **6c**

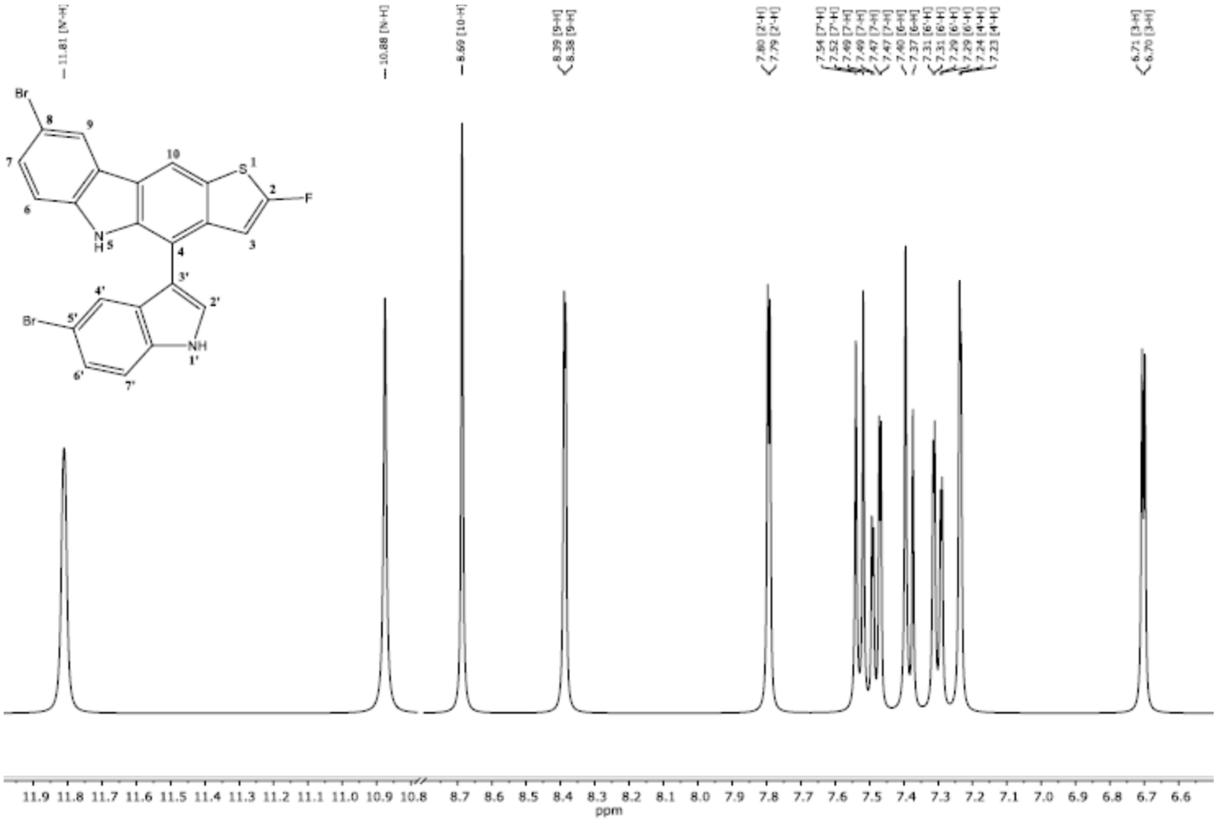

^{1}H NMR spectrum of **6d**
